# The transcriptional response to the olive fruit fly (*Bactrocera oleae*) reveals extended differences between tolerant and susceptible olive (*Olea europaea* L.) varieties

**DOI:** 10.1371/journal.pone.0183050

**Published:** 2017-08-10

**Authors:** Filomena Grasso, Mariangela Coppola, Fabrizio Carbone, Luciana Baldoni, Fiammetta Alagna, Gaetano Perrotta, Antonio J. Pérez-Pulido, Antonio Garonna, Paolo Facella, Loretta Daddiego, Loredana Lopez, Alessia Vitiello, Rosa Rao, Giandomenico Corrado

**Affiliations:** 1 Dipartimento di Agraria, Università degli Studi di Napoli “Federico II”, Portici (NA), Italy; 2 Centro di Ricerca per l’Olivicoltura e l’Industria Olearia, Consiglio per la Ricerca in Agricoltura e l’Analisi dell’Economia Agraria (CREA), Rende (CS), Italy; 3 Institute of Biosciences and Bioresources (IBBR), CNR, Perugia, Italy; 4 Trisaia Research Center, Italian National Agency for New Technologies, Energy and Sustainable Economic Development (ENEA), Rotondella (MT), Italy; 5 Departamento Biología Molecular e Ingeniería Bioquímica, Universidad Pablo de Olavide, Sevilla, Spain; University of Crete, GREECE

## Abstract

The olive fruit fly *Bactrocera oleae* (Diptera: Tephritidae) is the most devastating pest of cultivated olive (*Olea europaea* L.). Intraspecific variation in plant resistance to *B*. *oleae* has been described only at phenotypic level. In this work, we used a transcriptomic approach to study the molecular response to the olive fruit fly in two olive cultivars with contrasting level of susceptibility. Using next-generation pyrosequencing, we first generated a catalogue of more than 80,000 sequences expressed in drupes from approximately 700k reads. The assembled sequences were used to develop a microarray layout with over 60,000 olive-specific probes. The differential gene expression analysis between infested (i.e. with II or III instar larvae) and control drupes indicated a significant intraspecific variation between the more tolerant and susceptible cultivar. Around 2500 genes were differentially regulated in infested drupes of the tolerant variety. The GO annotation of the differentially expressed genes implies that the inducible resistance to the olive fruit fly involves a number of biological functions, cellular processes and metabolic pathways, including those with a known role in defence, oxidative stress responses, cellular structure, hormone signalling, and primary and secondary metabolism. The difference in the induced transcriptional changes between the cultivars suggests a strong genetic role in the olive inducible defence, which can ultimately lead to the discovery of factors associated with a higher level of tolerance to *B*. *oleae*.

## Introduction

The olive fruit fly, *Bactrocera oleae* (Diptera: Tephritidae), is arguably the single largest threat of cultivated olive (*Olea europaea* L.). Since olive domestication, *B*. *oleae* is the most devastating pest in the Mediterranean basin and the recent invasion of California suggests that the fruit fly follows its host [1, 2]. Phytophagous larvae feed exclusively on olive fruits [3]. Depending on climate conditions, the fruit fly can produce severe crop damage and significant economic loss in the whole olive sector [4].

Adult females pierce the olive and lay eggs under the exocarp. Hatched larvae progressively consume the olive pulp, causing tissue loss, premature fruit drop and reduction of oil yield. Moreover, fly infestation increases olive oil acidity and peroxide value, as well as musty and earthy off-flavours, extensively reducing oil quality (e.g. downgrading extra virgin olive oil to less valuable categories). Indirect effects are mainly due the presence of necrotic areas and microorganisms in feeding tunnels [5–9]. The conventional *B*. *oleae* management relies on chemical insecticides and/or traps [4]. The olive fly, as many other pests, can acquire resistance to pesticides [10], increasing the need for more effective biological or integrated control methods [11–13].

Considering the unrivalled olive oil-health benefits, research on the olive-fruit fly interaction has the long-term potential to influence not only the olive oil production but also the health-promoting properties of the olive oil. The economic impact of the *B*. *oleae* does not match the research efforts aimed to examine the interaction between the olive and its key enemy. For instance, compared to other biotic stresses, very few studies shed light on the mechanisms underlying olive defence and resistance at molecular level [14, 15].

Olive varieties can have different levels of tolerance to the olive fruit fly [4, 16]. In a comparison of different cultivars, the percentage of infestation ranged from less than 10% to up to 31% [16]. This difference is significant because a percentage of exit holes lower than 10% is compatible with a production of high quality olive oil in absence of chemical control methods, if adequate harvesting and storing procedures are followed [7]. A different tolerance to the olive fruit fly is evident not only comparing cultivars of different origins but also analysing regional germplasm [16, 17]. For instance, among the twenty varieties that constitute the olive germplasm of the Campania region (Southern Italy) [18], the ‘Ortice’ and ‘Ruveia’ are reported to be highly susceptible and tolerant, respectively [19].

The basis of the different tolerance to the olive fruit fly is expected to be complex [20]. It may rely on mechanical barriers (e.g. aliphatic waxes), chemical factors (e.g. oleuropein, cyanidine), morphological characteristics (e.g fruit size) and their combination. Similarly, the relative prominence and the contribution of these factors are yet to be fully clarified [21–25]. Moreover, the mechanism underlying the different levels of tolerance to the *B*. *oleae* has never been studied at molecular level. Finally, it is not known whether those features are constitutively expressed or induced by the fruit fly feeding.

To address these points, we studied the molecular response of the drupe in two olive cultivars with different levels of tolerance to the fruit fly. To this aim, we first generated a catalogue of more than 80.000 unigenes by next-generation pyrosequencing and then developed a microarray layout as affordable tool for functional genomics in olive. Our study provides insights into the molecular reaction of the drupe to larva feeding and illustrates the complexity and the differences of the drupe defence response of varieties with different levels of fruit fly susceptibility.

## Material and methods

### Plant material

Two olive varieties were studied because of their contrasting tolerance to *B*. *oleae*, ‘Ortice’ (susceptible to the olive fruit fly) and ‘Ruveia’ (tolerant) [19]. Olives were harvested from field plants at the Azienda Agricola Regionale Sperimentale “Improsta” (Eboli, Salerno) when at the Jaen Ripening Index (JRI) 2 [26]. The percentage of olive fly attack was calculated on 300 drupes per variety (10 replicated groups of 30 randomly collected drupes). Statistical differences were evaluated with a Student *t*-test. For molecular studies, olives with visible symptoms of pathogen attack as well as those with fly exit holes were discarded. Olives at different attack stages (punctured, with II or III instar larva, or pupae) were pooled and used with undamaged olives for massive parallel sequencing, in order to create a comprehensive repertoire of genes modulated by *B*. *oleae*. For the study of differential gene expression by microarray analysis, we used for each cultivar drupes with feeding II or III instar larvae and undamaged controls. For massive sequencing and for microarray analysis, olives were sliced under a light microscope to remove the larva. At this stage, drupes with sterile stings and dead/inactive larvae were also discarded. Slices were frozen in liquid nitrogen and stored at -80°C until use.

### 454 sequencing

Total RNA was isolated from 200 mg of drupe with the RNeasy Plant Mini Kit (Qiagen Hilden, Germany) and treated with DNase I (Ambion, Austin, TX, USA). Three replicates, each consisting of five olives, were used for each biological condition (punctured olives, olives with feeding II or III instar larva, olives with pupae and undamaged olives). cDNAs were synthesized using the SMART PCR cDNA Synthesis kit (Clontech, Palo Alto, CA, USA). First strand synthesis was performed using eight μg of total RNA as described [27]. Double stranded cDNAs were purified using the QIAquick PCR purification kit (Qiagen, Hilden, Germany) and quantified with a fluorimeter (Victor 2, Perkin Elmer, Wellesley, MA, USA). To estimate cDNA quality and fragment length, samples were separated on a 1.5% agarose gel. cDNA from olives characterized by different stages of fly attack were pooled together to obtain a representative ‘damaged’ sample. Two cDNA libraries (damaged and undamaged olives) per cultivar (‘Ortice’ and ‘Ruveia’) were sequenced. Approximately five μg of cDNA were sheared into small fragments via nebulization. The four shotgun cDNA libraries were sequenced using a 454 GS FLX+ Titanium Sequencer (Roche Diagnostics Corporation, Basel, Switzerland). The adaptor-trimmed 454 reads were assembled using the GSAssembler Software (Roche Diagnostics Corporation, Basel, Switzerland). To annotate unigenes (contigs and singletons), a blastX-based similarity search (e-value ≤ 1e-5) was used, querying the NCBI non-redundant (nr) database. We mapped the GI identifiers (http://www.ncbi.nlm.nih.gov/) of the best blastX hits to the UniprotKB protein database (http://www.uniprot.org/) in order to extract Gene Ontology (GO, http://www.geneontology.org/) and KEGG orthology (KO, http://www.genome.jp/kegg/) terms.

### Microarray preparation and hybridization

CustomArray^™^ (CustomArray Inc., Bothell, WA, USA) microarrays containing over 90k probes were built at the ENEA—Trisaia Research Center (Rotondella, MT, Italy). Probes were designed using the ProbeWeaver software (CustomArray Inc., Bothell, WA, USA), based on the 454 pyrosequencing results of four cDNA libraries obtained from damaged and undamaged drupes of the ‘Ortice’ and ‘Ruveia’ varieties.

The chip layout consists of 61,825 olive probes (60,706 non-redundant) out of the 87,720 sequences of the pooled library. The layout includes around 20.000 quality control spots. Probes were 35- to 40-mers with a melting temperature of 70–75°C and were synthesized on microarrays through the CustomArray Synthesizer^™^ (CustomArray Inc., Bothell, WA, USA).

Total RNA was isolated from 200 mg of finely ground olive tissue. A purification step was performed in a 1:1:2 (v/v/v) solution of phenol, chloroform and RNA Extraction Buffer (1 M Tris-HCl pH 8.5, 5 M NaCl, 0.5 M EDTA pH 8.0, 10% SDS) twice, followed by a chloroform purification. RNA isolation from the aqueous phase and quality control were performed as described [28]. Samples with a 260/280 nm absorbance ratio higher than 1.8 and a 260/230 nm absorbance ratio higher than 2.0 were used for subsequent experiments. For each biological condition, three independent samples (i.e. from different trees) were obtained as pools of three to five independently extracted technical replicates. Two μg of total RNA were retrotranscribed using the RNA ampULSe Amplification and Labeling Kit for CustomArray^™^ microarrays (Kreatech Biotechnology). cRNA labeling was carried out with the Cy5-ULS (Cyanine-Universal Linkage System). Unincorporated dye was removed with a KREApure purification column (Agilent). Labeling yield and quality were assessed using a NanoDrop 1000 (Thermo Scientific). cRNAs were fragmented for 20 min at 95°C in 200 mM Tris-Acetate (pH 8.1), 500 mM potassium acetate and 150 mM molybdenum acetate. The fragmented Cy5-cRNAs were added to the hybridization solution (6X SSPE, 0.05% Tween-20, 20mM EDTA, 25% formamide, 100 ng/μl salmon sperm DNA, 0.04% SDS) and poured in the hybridization chambers of the pre-hybridized microarrays, following the CustomArray^™^ Hybridization protocol. After an overnight incubation at 45°C in a rotating rotisserie oven, microarrays were treated with six washing steps: firstly, in 6X SSPE and 0.05% Tween-20 for 5’ at 45°C, then with 3X and 0.5X SSPE and 0.05% Tween-20 for 1’ at room temperature, with 2X PBS and 0.1% Tween-20, and finally twice with 2X PBS for 1’ at room temperature.

For the validation of the microarray results, Real time PCR was carried out as described [14]. Primers and their main features are reported in S1 Table.

### Microarray data analyses

Microarray slides were scanned with the GenePix Pro microarray scanner (Axon Instruments) and data processed with the CustomArray^™^ Microarray Imager software (CustomArray Inc., Bothell, WA, USA). A maximum threshold of 0.20 for the coefficient of variance (of the spots corresponding to identical probes) was applied to control intra-chip hybridization variability. The correlation among the three technical replicates for each experimental condition was assured by a minimum Pearson coefficient (R) of 0.99. Stripping and preparation of the microarrays for re-hybridization was performed twice, considering the three technical replicates, according to the manufacturer’s instruction (CustomArray Inc., Bothell, WA, USA). Raw values were normalized on the median of the intensities by the ProbeWeaver software (CustomArray Inc., Bothell, WA, USA) using the quantile algorithm. Pairwise analysis of differential expression was assessed with a Welch's t-test, followed by a Bonferroni-Hochberg FDR adjustment with a cut off p-value of 0.05, using R [29]. To filter out weakly expressed sequences, we calculated the average and standard deviation of the expression value of all empty and negative controls and set as threshold the mean value plus two times the standard deviation [30]. Of the filtered, significantly differentially expressed probes, only those with greater than 2-fold increase or 2-fold decrease in expression compared to the control condition were used for further analysis. Principal Component Analysis was performed in R [29]. K-means clustering analysis of the differentially expressed genes (DEGs) was carried out with the Multi Experiment Viewer (MeV) software [31]. Dataset grouping was performed with the average linkage clustering methods based on Euclidean distances. Different number of clusters (k fixed from 8 to 16) were compared and k = 10 was selected to maximize partitioning and to avoid empty or very small groups. The corresponding expression graphs were visualized with MeV. We followed the Minimum Information About a Microarray Experiment (MIAME) guidelines for microarray analysis and verification [32].

### Functional annotation of differentially expressed genes

Functional annotation was carried out by sequence analysis using the Blast2GO software [33]. Briefly, for each sequence corresponding to a differentially expressed probe, a blastX similarity search (e-value < 1e-6) against the non-redundant (nr) NCBI database (Non-redundant GenBank CDS translations including RefSeq, PDB, SwissProt, IR and PRF) was performed to retrieve a maximum of 20 top homologous hits per query. The GO-term mapping and annotation were retrieved using NCBI as well as non-redundant reference protein database (PSD, UniProt, RefSeq, GenPept, PDB Full Gene Ontology DB). Sequences with a blast hit that could not be mapped and annotated were then blasted (blastX) against the *Arabidopsis thaliana* protein sequences and the *Oryza sativa* protein sequences database. Additional annotations (e.g. the recovery of implicit “Biological Process” and “Cellular Component” GO-terms from “Molecular Function” annotations) were implemented using ANNEX 5.0 [34]. Completion of the functional annotation with protein domain information was performed with InterProScan 5.0. A plant GO-Slim reduction was carried out to summarize the functional content of the dataset. To complete the functional annotation Sma3s was also used [35]. Plant taxonomic division from UniProt was selected as source of annotations, and the transcriptomic sequences were enriched with keywords, InterPro domains and motifs, and GO terms. We generated multi-pie chart summary to present meaningful GO identifiers that are at hand yet not excessively general. When possible, sequences that could not be mapped were named (but not annotated) after a tblastX search against the nr NCBI database to identify sequences similar to the query based on their coding potential.

## Results

The already reported different susceptibility to *B*. *oleae* of the two varieties was evaluated by measuring the percentage of attack at the time of harvesting. The susceptible variety ‘Ortice’ had an attack index 2.5 times higher than the ‘Ruveia’ (p<0.01). At sample harvest, the infestation level was 13.3% in ‘Ortice’ and 5.3% in ‘Ruveia’.

Pyrosequencing of the four cDNA libraries from damaged (pool of different stages of fly attack) and control undamaged fruits of ‘Ortice’ and ‘Ruveia’ generated 695,511 reads, with an average length of 361 bp (N50: 421 bp; N75: 359 bp) (Table 1).

**Table 1:** Overview of the raw data output from the 454-FLX Titanium sequencing.

Sample	Total bases	Reads	Mean length (bp)
‘Ortice’ control	63,611,956	173,118	367
‘Ortice’ damaged	73,464,937	197,782	371
‘Ruveia’ control	52,792,933	146,765	360
‘Ruveia’ damaged	61,220,898	177,846	344
Total	251,090,724	695,511	

We assembled the raw reads from the four libraries together. More than 80% of raw sequences were included in the assembly and 72,662 remained as singleton (Table 2).

**Table 2:** Main indices and statistics of the assembly.

Samples in assembly	Reads in Assembly (%)	Contigs		Singletons	
		Number	Average length (bp)	Number	Average length (bp)
‘Ortice’ control ‘Ortice’ damaged ‘Ruveia’ control ‘Ruveia’ damaged	570,878 (82.93)	15,058	884	72,662	333

The assembly yielded 15,058 contigs, with an average length of 884 bp and 78% of the sequences longer than 500 bp (Panel A in S1 Fig). Approximately 40% of the contigs were composed of 2–10 reads. The majority of the contigs (28%) were low-coverage (6–10 reads) and around 5% were high-coverage (> 100 reads). The unigenes list and their annotation is reported in S2 Table. An overview of the putative functions of the unigenes, based on a blastX-similarity search annotation, is presented in S2 Fig. About 55% of the unigenes matched to a protein product. The remaining 39,657 had no function assigned (Panel A in S2 Fig). The best blast-hits belonged to *Vitis vinifera* (44%), *Populus trichocarpa* (15%) and *Ricinus communis* (5%) (Panel A in S2 Fig). Approximately 24.4% and 6.1% of the unigenes was assigned to at least one Gene Ontology (GO) and KEGG orthology (KO) terms, respectively. Unigenes were distributed in 14 GO-terms for the Biological Process ontology, 9 for Cellular Component and 10 for Molecular Function. Metabolic process sub-category, consisting of 11,167 genes, was dominant in biological process. Binding and cell part subcategories, consisting of 13,820 and 8,292 genes, were dominant in molecular function and cellular component, respectively. A considerable number of genes was included in cellular process, catalytic activity and intracellular sub-categories (Panel B in S2 Fig). To identify main biological pathways, we mapped the annotations to reference canonical pathways in the KEGG database by using KO identifiers (i.e. a classification of orthologous genes defined by KEGG). The 5,355 KO allocated genes were assigned to 265 KEGG categories. The most abundant processes/metabolic pathways were “biosynthesis of secondary metabolites” (185 unigenes); “microbial metabolism in diverse environments” (89); “spliceosome” (74); “biosynthesis of plant hormones” (72); “ribosome” (61); “RNA transport” (61) and “protein processing in endoplasmic reticulum” (60) (Panel B in S2 Fig).

### Differential gene expression

To profile the variation in gene expression of olives during *B*. *oleae* feeding and to characterize the differences between the two varieties with contrasting susceptibility to the olive fruit fly, unigenes were used to build a CustomArray^™^ (CombiMatrix Corporation). The chip layout consists of 61,825 olive probes out of the 87,720 assembled sequences (Panel B in S1 Fig). The DNA microarrays were then hybridized with labeled cRNA prepared from undamaged olives of the varieties ‘Ortice’ or ‘Ruveia’ and corresponding samples with feeding larvae.

**Fig 1:**
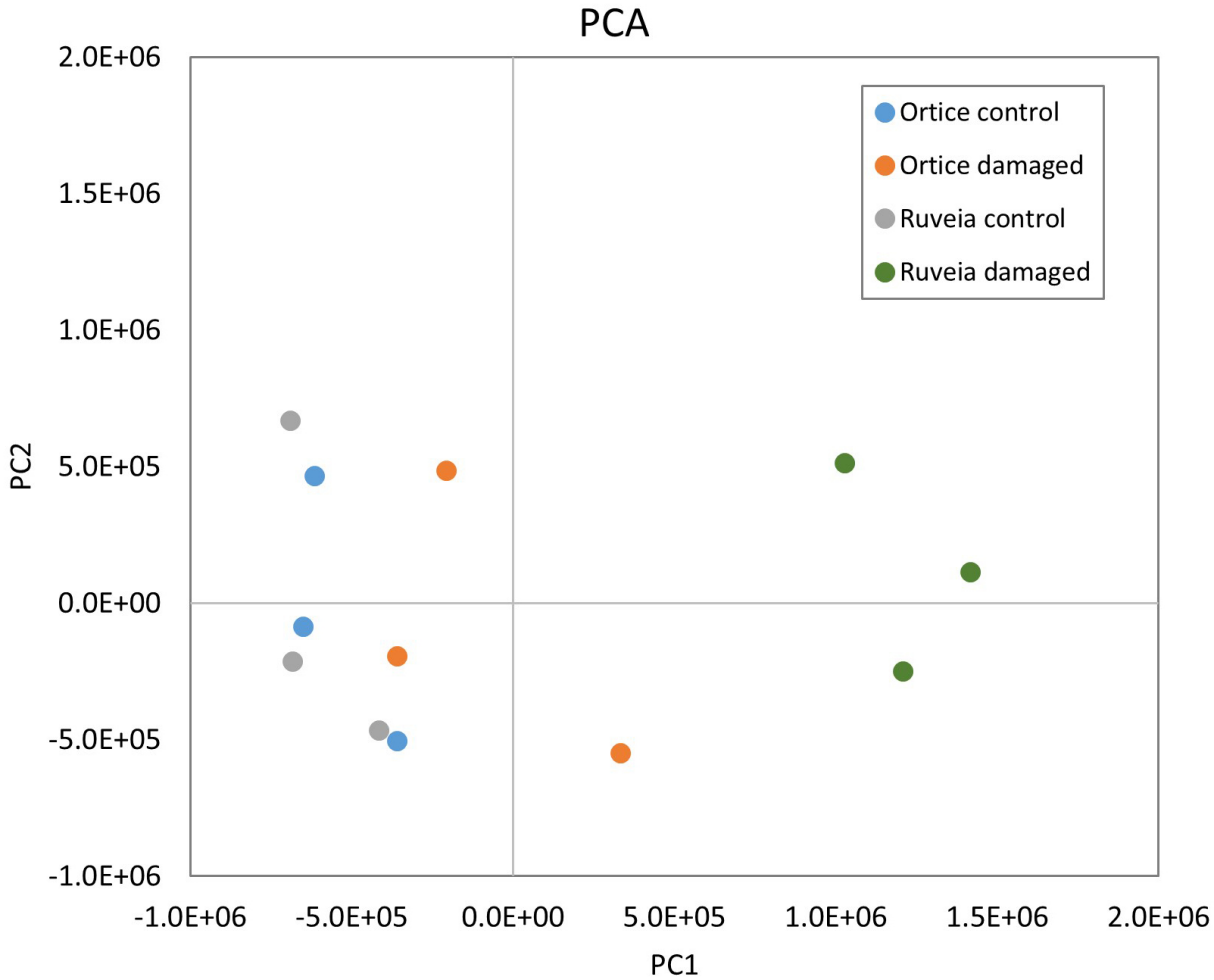
Principal Component Analysis of the normalized microarray data of the olive probes. The percentage of the total variation explained by the first two principal components (PC1 and PC2) was 46.0 and 14.1, respectively. The PCA plot shows separation of the four experimental groups, indicated by different colors, along the first component and clustering of the three biological replicates, represented by a dot, within these groups.

After normalization (S3 Fig), the expression data relative to all the olive probes were analysed by PCA to explore the variation among the different conditions based on gene expression states (Fig 1).

The variability among the different conditions and biological replicates could be summarized and visualized in two dimensions. The first component, which accounted for 46.0% of total variance, appears to represent the overall induction due to the fruit fly, as it discriminates the different biological response of the two varieties. The second component, which explained 14.1% of the variance, mainly discriminated the biological replicates for each condition. Because of a clear *B*. *oleae* effect, the limited difference among the control conditions of the two cultivars and the dispersion of the biological replicates, we retained all conditions and replicates for further analysis.

To identify differentially expressed probes, we applied the following selection criteria: a *p*-value <0.05 (Welch t-test, followed by a Bonferroni-Holchberg FDR correction for multiple testing) and a |log_2_Ratio|> 1. The number of differentially expressed genes, after removal of duplicates, is reported in Table 3.

**Table 3:** Differentially expressed genes (DEGs) in pairwise comparisons.

	Upregulated	Downregulated
‘Ruveia’ control vs ‘Ortice’ control	23	7
‘Ortice’ damaged vs ‘Ortice’ control	17	75
‘Ruveia’ damaged vs ‘Ruveia’ control	1071	1528
‘Ruveia’ damaged vs ‘Ortice’ damaged	714	537

DEGs and their annotation are listed in S1 File. The heatmap illustrates a weak linkage among the four conditions (S4 Fig). To validate the microarray results, the expression of six genes (four differentially expressed genes and two non-affected by *B*. *oleae*) was analyzed by Real Time-PCR. The results were consistent to the microarray data (S5 Fig).

**Fig 2:**
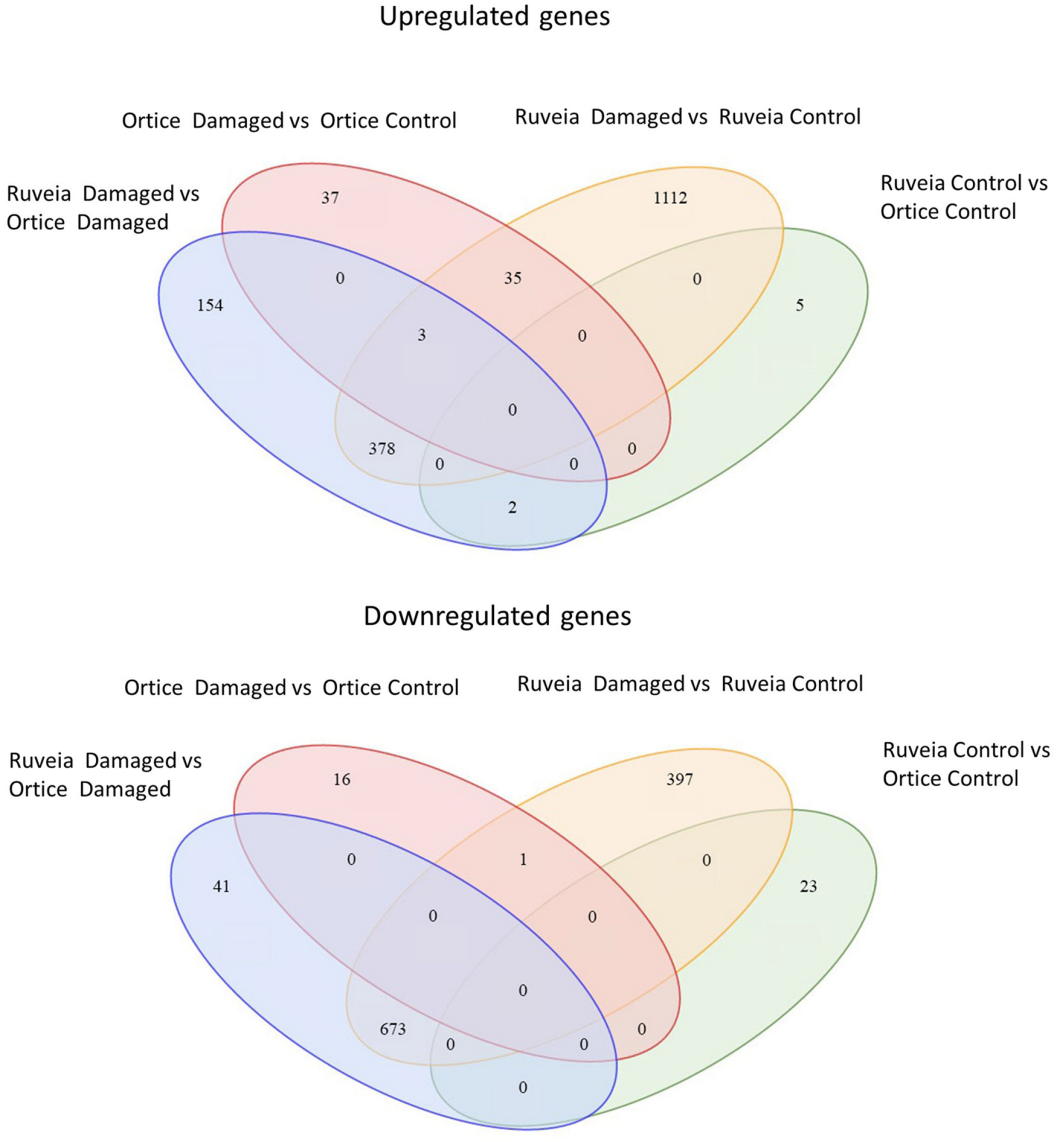
Venn diagram of the differentially expressed genes. The intersecting portions of the Venn diagrams report the number of common genes among the different comparisons between the two varieties (‘Ruveia’ and ‘Ortice’) in the two experimental conditions (control and *B*. *oleae* damaged).

The smallest difference, in terms of differentially expressed genes, was present between the undamaged olives of the two cultivars. The response to the *B*. *oleae* feeding of the susceptible cultivar ‘Ortice’ also involved a limited set of genes and the majority (77%) were down regulated. The response of the more tolerant variety ‘Ruveia’ involved the highest number of both up-regulated and down-regulated genes. When attacked, olives of the ‘Ruveia’ cultivar differentially expressed more than 1000 genes compared to attacked drupes of the ‘Ortice’. To investigate the commonality between the olive varieties and their response, DEGs in the pairwise comparisons were matched. The response of ‘Ruveia’ is highly specific, as indicated by the limited number of common genes. Around half of the genes down-regulated in ‘Ortice’ were also down-regulated in ‘Ruveia’ (Fig 2).

The majority of the genes overexpressed in ‘Ruveia’ after *B*. *oleae* attack were also upregulated when comparing the damaged ‘Ruveia’ and ‘Ortice’ drupes. Overall, the comparison of the various response to the B. oleae indicated that two varieties have a markedly different reaction.

### Transcript clustering

**Fig 3:**
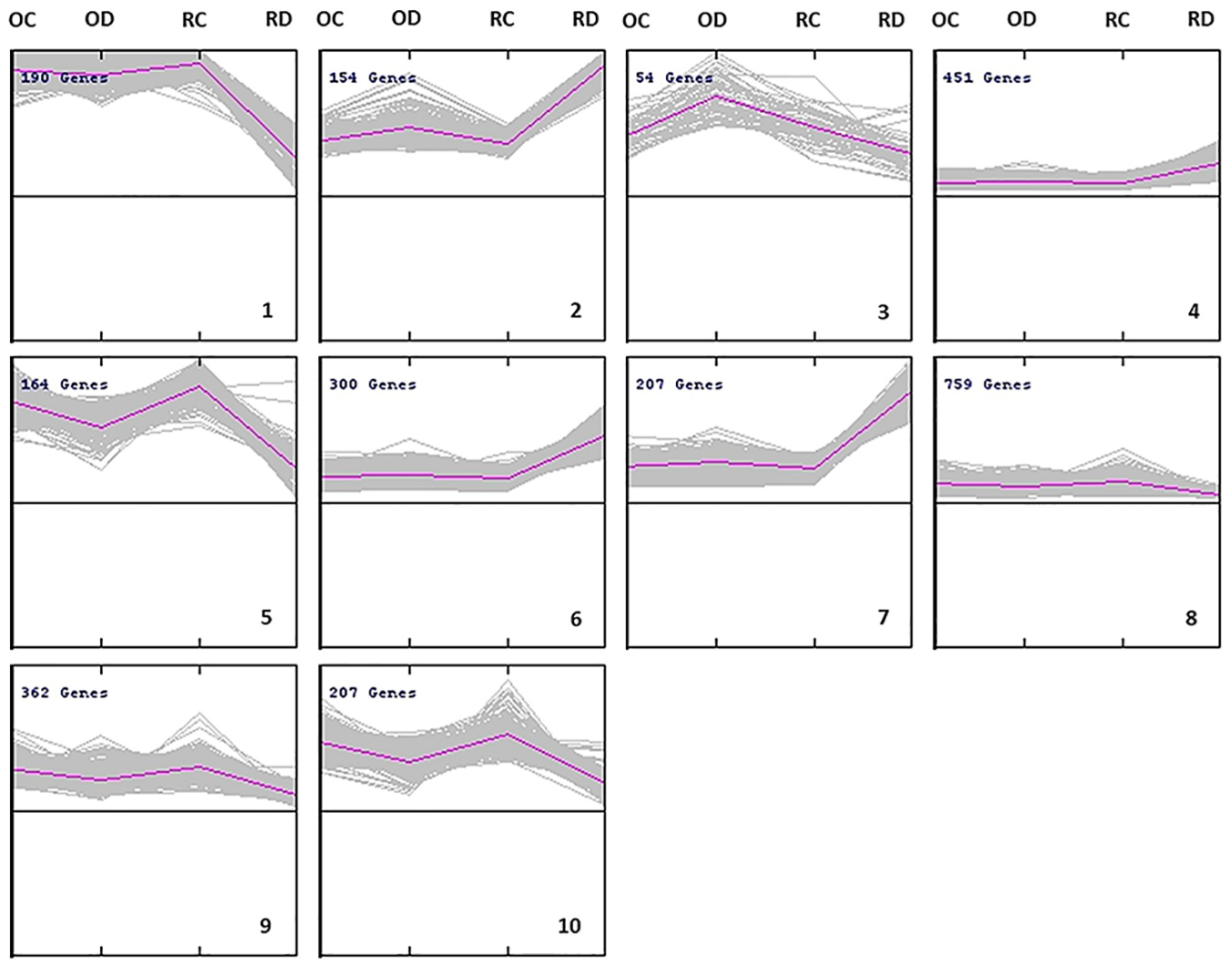
K-means clustering of the differentially expressed gene (FDR< 0.05 and |log_2_ Ratio| > 1). In each box, grey lines represent genes, whose expression level vary in the four experimental conditions namely, ‘Ortice’ undamaged (OC), ‘Ortice’ damaged (OD), ‘Ruveia’ undamaged (RC) and ‘Ruveia’ damaged (RD). A pink line represents the average expression profile of all genes in each cluster. In the top-left corner, it is reported the total number of genes in the cluster. A progressive cluster (K) number is indicated in the bottom-right corner.

Considering the limited overlap of the molecular response of the two varieties, the expression level of the statistically significant genes in at least one of the four comparisons was processed by K-means clustering analysis to infer possible co-expressed or co-regulated genes (Fig 3).

**Fig 4:**
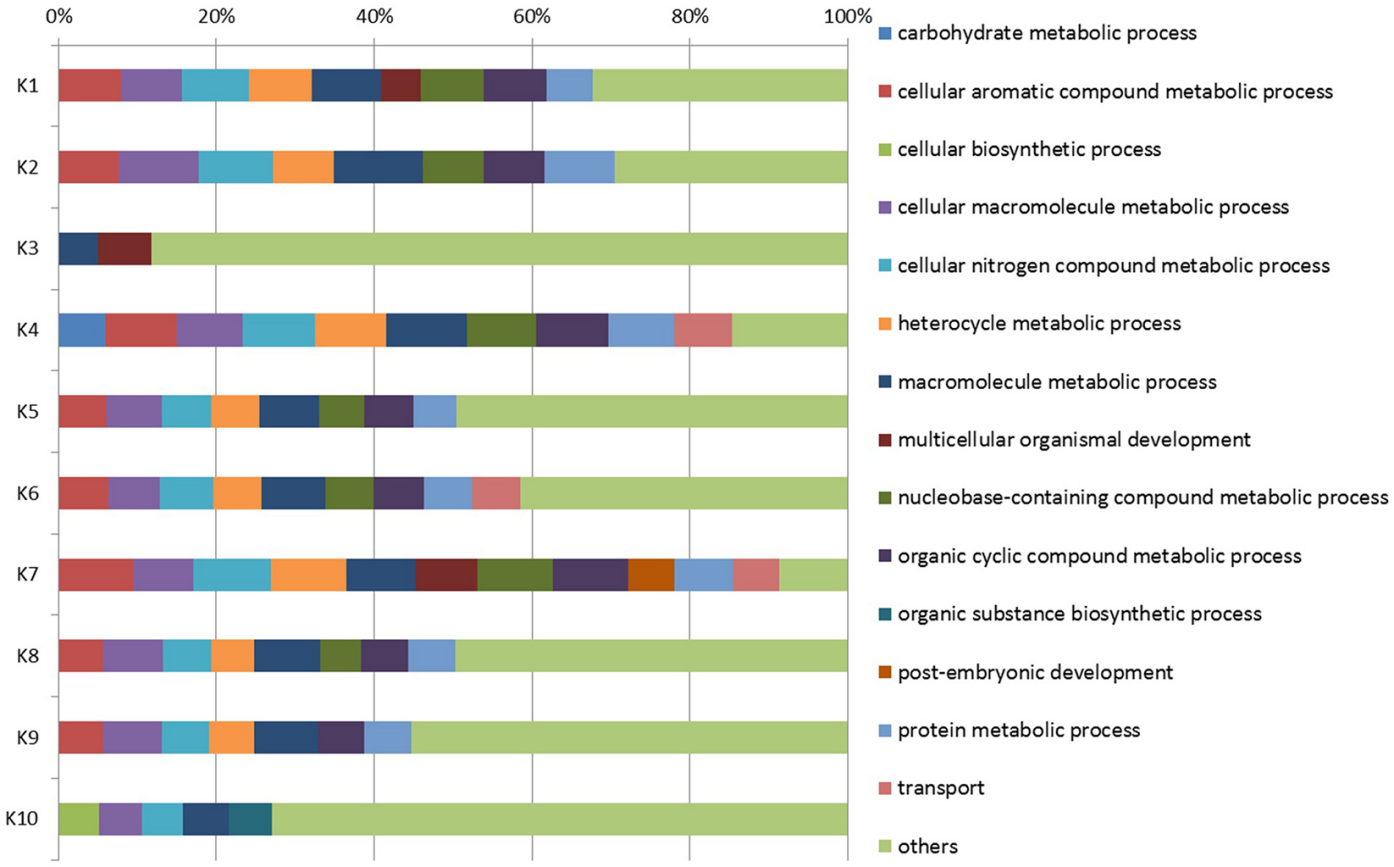
Functional categories of the clusters of the DEGs (FDR< 0.05 and |log_2_ Ratio| > 1). Each horizontal bar refers to the clusters (numbered from K1 to K10; see Fig 3) deriving from K-means clustering. Bars are partitioned into colored segments whose length represents the relative number of sequences for each GO term (level 4; BP; sequence cut-off: 5%).

This analysis allowed to group the 2848 DEGs in 10 clusters (S3 Table). K-means clustering indicated that it was not possible to identify a cluster in which genes are overexpressed in the two attacked varieties with a similar pattern, further indicating a very limited overlap between the responses of the two olive varieties. The GO annotation (level 4) in the “Biological Process” domain of the genes belonging to the clusters (sequence cut-off 5%) is reported in Fig 4.

To ease the comparison, the graph reports, for each cluster, the different GO categories in relative terms. The annotation indicated that K7 and K4 are the most complex clusters because they include, respectively, 12 and 11 out of the 15 considered GO terms. Taking as reference the average expression pattern, clusters K7 and K4 mainly comprise genes that are, respectively, highly and mildly up-regulated exclusively in the damaged drupes of the more tolerant cultivar ‘Ruveia’. Similarly, genes belonging to clusters K1 and K6, specifically modulated in ‘Ruveia’ drupes, were annotated with a high number of GO categories.

The most present GO terms in the different clusters were related to chemical reactions and pathways involving macromolecule, such as “macromolecule metabolic process” (present in all the ten clusters), “cellular macromolecule metabolic process” and “cellular nitrogen compound metabolic process” (present in nine clusters). K4 is the only cluster containing transcripts related to “carbohydrate metabolic processes” while the “post-embryonic development” term was exclusively present in K7. This analysis indicated the degree of specificity of the molecular events and biological processes characterizing the response of the tolerant cultivar ‘Ruveia’ to *B*. *oleae* attack.

### Expression analysis of the ‘Ruveia’ response to fruit fly

**Fig 5:**
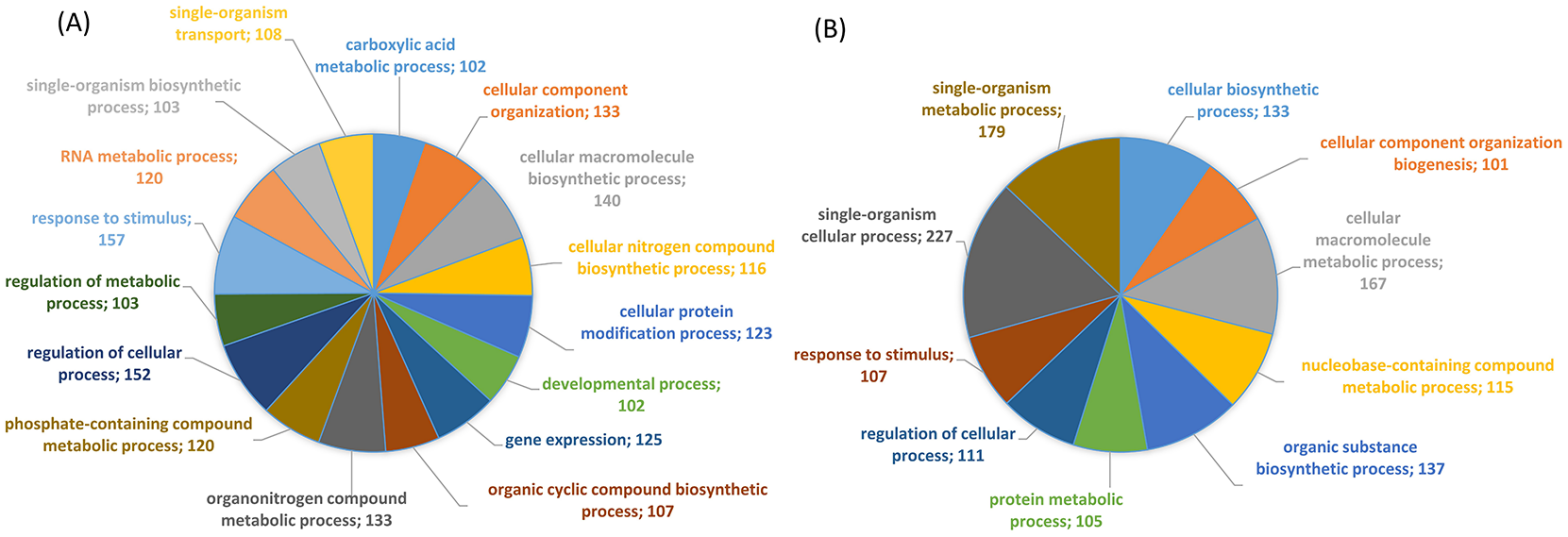
Multilevel distribution of the differentially expressed annotated sequences by GO category. The lowest terms with a minimum of 100 annotated sequences are shown. For each category it is indicated the number of annotated sequences. (A) downregulated genes; (B) upregulated genes.

We used GO terms association (obtained by a sequence similarity search) to elucidate the biological objective to which the DEGs contribute in the response to the *B*. *oleae* feeding. GO analysis indicated that the transcriptional reconfiguration involved a range of biological processes (S6 Fig). To provide an optimal view of the dataset’s most relevant terms, a summary of the annotation results is presented as a multi-level pie that shows the lowest GO terms per branch that fulfil our annotation criterion weight (Fig 5).

The functional profile of both down and upregulated sequences indicated that the most abundant GO term categories were related to chemical reactions and pathways resulting in the formation of substances, including protein modification processes. Collectively, response to stress and to abiotic or biotic stimulus ranked the first most affected biological process for the down-regulated genes and at the eighth place for the up-regulated sequences.

Among the up-regulated genes involved in the response to stress, there were transcripts coding for proteins involved in signaling, including receptors and transcription factors, such as two leucine-rich repeat receptors, nine WD-repeat containing proteins, five WRKY transcription factors, and two rpm1-like proteins. The latter are essential regulator of plant defense and are typically associated to the resistance to pathogens [36]. Other up-regulated genes typically associated to pathogenesis included two PR proteins, one disease resistance response protein, one late blight resistance protein homologue and a NBS domain resistance protein. Among the genes that can directly deter insect growth, there were three beta-glucosidases. In fruits, beta-glucosidase activity plays an important role in the oleuropein metabolism, catalyzing its hydrolysis into a toxic glutaraldehyde-like structure that acts as defense mechanism against insects [37]. Moreover, four serine carboxypeptidase-like (SCPL) genes were up-regulated. In tomato, Arabidopsis and rice, some SCPL proteins are wound inducible and connected to jasmonic acid (JA) pathway [38, 39]. Other up-regulated genes possibly related to JA pathway include two phospholipases. Genes typically associated to abiotic stress were also up-regulated, such as two transcripts coding for dehydration-induced proteins, two heat-shock proteins and a bobber1–like protein. The ‘Ruveia’ response to *B*. *oleae* also included genes involved in phytohormone signaling such as those coding for proteins involved in auxin (i.e. auxin binding protein, auxin efflux carrier family protein, auxin response factor, indole-3 acetic acid amino-acid hydrolase), brassinosteroid (i.e. brassinosteroid insensitive 1-associated receptor kinase 1 like, bri1 suppressor 1 like) and ethylene signaling (i.e. four ethylene responsive transcription factors). Besides stress hormones, the plant perception and the signal transduction of *B*. *oleae* infestation seems to involve also other stress-related cellular messengers such ROS and calcium, as indicated by the up-regulation of five calcium-dependent CBL-interacting serine/threonine protein kinases, a calcium transporting ATPase plasma membrane, four glutaredoxin, and one oxoglutarate genes.

KEGG pathway map analysis of annotated enzymatic activities indicated that purine metabolism ranked first as number of sequences. Among other physiological processes, variation in purine metabolism associates to fruit ripening and to uracil salvage activity during senescence. Moreover, plants produce toxic secondary metabolites from pyrimidines that act as defense compounds [40]. The “Biosynthesis of antibiotics” KEGG reference pathway ranked first considering the number of enzymatic activities. This molecular interaction network diagram comprises an ample number of reactions that in plants are associated to terpenoid backbone biosynthesis, the shikimate pathway and the biosynthesis of secondary metabolites. Starch and sucrose metabolism ranked second for both the number of sequences and enzymatic activities.

### Differential expression analysis of the damaged drupes of the two varieties

To understand the inducible factors that can account for the different tolerance of the two varieties, we compared the gene expression levels between ‘Ortice’ and ‘Ruveia’ drupes when infested by *B*. *oleae*. The total number of DEGs was less than half compared with the response of the tolerant cultivar ‘Ruveia’ to the fruit fly. The proportion of the up-regulated genes (57%) was higher compared to the ‘Ruveia’ response, indicating that the different tolerance between the cultivars lies in the activation of a wide number of genes. In the Biological Process domain, differentially expressed sequences were assigned to a number of processes. At the GO level 2, the largest difference in ranking between the up-regulated and down-regulated sequences was for “multicellular organismal process”. On the opposite, “metabolic process”, in comparative terms, it is the most characterizing GO term in the down-regulated sequences (S7 Fig).

**Fig 6:**
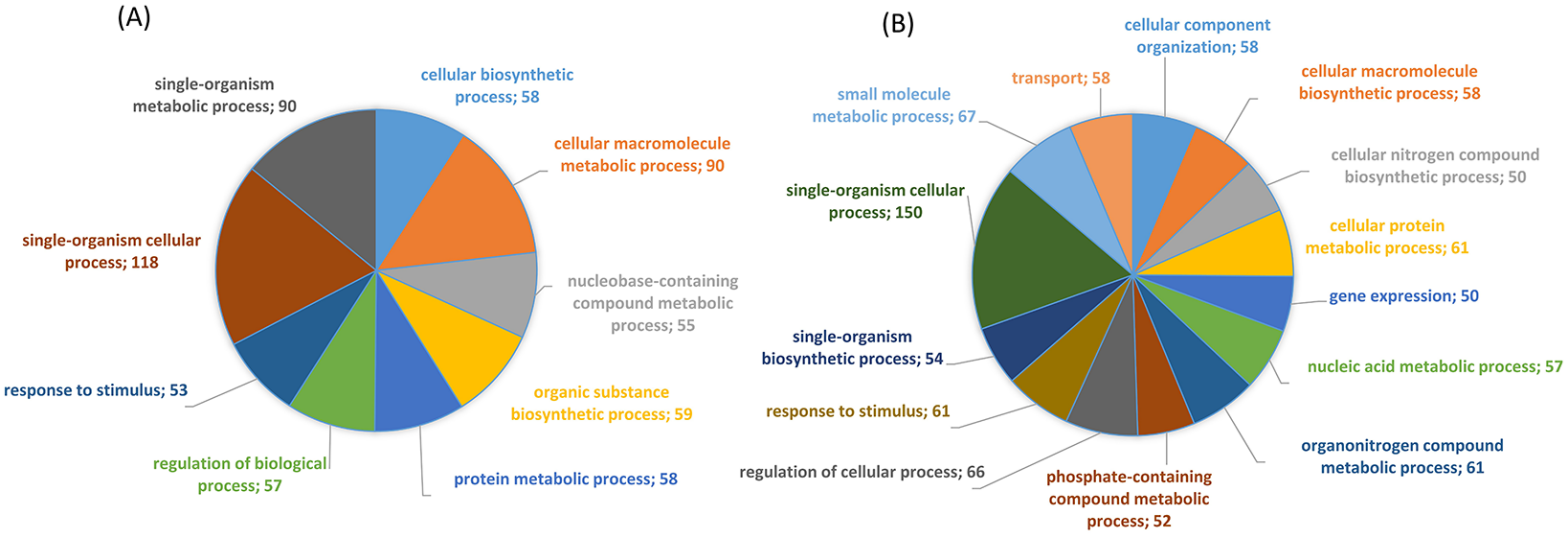
Multilevel distribution of the differentially expressed annotated sequences by GO category. The lowest terms with a minimum of 50 annotated sequences are shown. For each category, it is indicated the number of annotated sequences. (A) downregulated genes; (B) upregulated genes.

A summary of the GO annotation results for the down-regulated or the up-regulated sequences is presented as a multi-level pie (Fig 6).

The dominant GO term for both upregulated and downregulated sequences was “single-organism process”. “Response to stimulus” ranked fourth for the up-regulated sequences and ninth for the down-regulated sequences.

Among the up-regulated genes, eight sequences were associated to the GO term “response to biotic stimulus” such as a WRKY transcription factor 17, two putative serine/threonine kinases, and a Serine carboxypeptidase like. Genes coding for proteins putatively involved in phytohormone signaling included an auxin efflux carrier family protein and abri1 suppressor 1 like. These genes were also upregulated in ‘Ruveia’ cultivar in response to the olive fruit fly. In total, almost 95% of the genes up-regulated in the comparison between the attacked drupes of the two cultivars were also upregulated in the damaged fruits of the tolerant variety (‘Ruveia’). The data indicated that the different tolerance to the fruit fly in the two cultivars under investigation is mainly inducible.

## Discussion

The use of olive varieties that are highly tolerant to the fruit fly is an important element to reduce economic loss and use of chemical pesticides. Knowledge on the molecular aspects underlying the different resistance response to the fruit fly can assist the screening of more suitable genotypes and ultimately, contribute to the development of new integrated control strategies. For these reasons, we compared two olive cultivars with contrasting resistance levels to *B*. *oleae*. To this aim, we first generated a large collection of unigenes from our NGS data. The assembly did not include about 20% of raw sequences, a limited number considering the abundance of short repeats and the high level of heterozygosity of the olive [27, 41]. The reduced number of unassembled reads should have been also affected by the smaller number of cultivars and plant tissues under investigation in comparison with other works [41]. The 454 DNA sequencing method confirmed to be an effective technology in revealing the expression of a large number of genes in a non-model organism mainly because its longer read length [42].

We pooled the pyrosequencing samples derived from a number of conditions to generate a microarray based on the drupe sequence information and obtained a number of unigenes comparable to other works in olive [41]. Similarly, the percentage of annotation of our assembled dataset was comparable or higher to previous olive ESTs sequencing efforts [27, 41, 43]. The setup of a microarray allows analyses of gene expression and functional genomics studies in olive at a fraction of the cost of next generation sequencing. Moreover, microarray data are more computationally tractable and do not require an extended bioinformatics effort [44]. Our layout specifically focused on the drupe and was based on the widely used Combimatrix technology, hence extending currently available options [45, 46].

A key interest in this study was to compare the response of genotypes with different level of tolerance to the olive fruit fly. In absence of infestation, very little difference was noticed between the two cultivars. This is justified considering that we analysed plants present in the same environment, not only to reduce environmental variability but also to have the same *B*. *oleae* population level. A diverse expression profile between the tolerant and susceptible genotype was clearly observed only in the presence of larvae. Within a species, the scale and quality of response to herbivores as well as the accumulation of defence compounds may vary significantly [47–50]. In *Brassica oleracea*, there was a very little overlap between transcriptional responses of two varieties with contrasting level of resistance to the cabbage aphid (*Brevicoryne brassicae*), underlying that intraspecific variation in susceptibility to insect pests can be also explained by differences in induced transcriptional changes [51]. The here described strong difference between the tolerant and the susceptible variety also implies a significant genotype-specific response in olive. This would be consistent with the frequently reported genetic diversity and the large diversified phenotypic traits of the plant and of olive oil products [52]. The data suggest a strong relevance of the genetic component in the determination of the tolerant phenotype [53, 54] also because trees grew in the same environment using the same agronomic practices.

Plant defence pathways can be manipulated by also pathogens or pests [55, 56]. For instance, glucose oxidase, one of the principal components of *Helicoverpa zea* saliva, suppresses some induced resistance in *Nicotiana tabacum* by directly inhibiting the wound-signalling molecule jasmonic acid and/or by antagonizing its interaction with other signalling pathways [57]. In tomato, the spider mite Tetranychus evansi is able to suppress the induction of genes involved in the induced plant defences, such as proteinase inhibitors [58]. Evidence for the defence suppression by herbivores is not as large as for defence induction yet, it is conceivable that plant resistance mechanisms promote the selection of counter-adaptive mechanisms in biotic stressors, especially in compatible interactions involving monophagous pests [59]. Our data suggest that the successful colonization of drupes by *B*. *oleae* is likely to be due to a weak reaction in the more susceptible variety and that drupes of the more tolerant variety are a less favourable nourishment because of a more active and composite molecular response [60].

The gene expression in the attacked drupes of cv. ‘Ruveia’ identified a number of sequences directly and indirectly involved in the induced resistance mechanism. Plant response to herbivorous pest encompasses a number of mechanisms [61] and therefore, several molecular processes are activated by the drupe defence to the fruit fly. Upregulated genes include those involved in the defence mechanisms against biotic stress, both wounding and pathogen attack, as well as abiotic stress, such as drought and high or low temperature. The overexpression of genes involved in different defense pathways suggests that the inducible response to *B*. *oleae* larvae has a degree of overlap with the response to pathogens, which was also noted for the interaction between olive and *S*. *oleaginea* [62]. Moreover, our data is consistent with other research works that indicated that beta-glucosidases are important element of the olive defense to *B*. *oleae* [14, 15, 37]. Beta-glucosidases promote the formation of toxic glutaraldehyde-like structure from oleuropein, a phenolic compound that has been linked to the different susceptibility of olive cultivars [16]. DEGs were also involved in the production or response to phytohormones and molecules involved in pest response (e.g., jasmonic acid and ROS).

Besides genes associated to stress resistance, the Gene Ontology analysis suggested that biological processes related to secondary metabolism, cation transport and transmembrane transport are also affected, giving reasons to believe that numerous changes in plant primary metabolism should occur in response to larva feeding [63]. Recently, it has been reported that *B*. *oleae* infestation causes significant changes in mineral elements (such as P, K, Fe and Mg) in fruits [23]. Also abiotic stress, such as a moderate drought, affects different metabolites during fruit development, including terpenes [64]. Collectively, the existing molecular and metabolomics data suggest that the defensive reaction of the tolerant cultivar to the persisting feeding of the *B*. *oleae* comprises a larger than anticipated metabolic reprogramming in infested tissues, yet to be fully described [7, 65].

## Conclusions

The *B*. *oleae* feeding influences pathways with a known role in defence, oxidative stress responses, and genes involved in plant structure and metabolism. Defence against the long-lasting fruit fly larva feeding is a complex trait and involves multiple molecular mechanisms. The complexity of the drupe response suggests that a number of features, metabolites and signalling pathways effectively limit the fruit fly infestation. An additional level of complexity derives from the marked genotype-specific difference present in olive that is suggested by our study [66]. The extended gene expression difference between the tolerant and susceptible olive varieties under investigation also indicated that it would be possible to identify genetic factors that are associated with a higher level of tolerance to *B*. *oleae*.

## Supporting information

S1 Fig Length distribution of the assembled sequences (A) and of those used for the microarray (B). (TIF)

S2 Fig Functional annotation of the assembled unigenes. A) Distribution of the unigenes according to the presence (matched) or absence (non-matched) of hits retrieved from the NCBI database. The pie of the pie chart illustrates the relative distribution of the best-BlastX hits according to the plant species. B) Classification of the unigenes in the GO domains Biological Process, Cellular Component and Molecular Function. For each domain, the table reports the GO-terms (category) ranked in decreasing order according to the number of sequences (singleton/TCs). The bar chart illustrates for each GO-term the relative amount in relation to the number of sequences annotated in the GO-domain and the total number of sequences assembled.
(TIFF)

S3 Fig Box plots of signal intensity after quantile normalization for each microarray hybridization. Legend: Ort: ‘Ortice’; Ruv: ‘Ruveia’; S: control condition; T: test condition; Roman numbers denote, per each condition, the biological replicate.
(TIF)

S4 Fig Heatmap of the differentially expressed genes. The heatmap shows the relative expression level of the DEGs in the four experimental comparisons. 1: ‘Ruveia’ Test vs ‘Ruveia’ Control condition; 2: ‘Ruveia’ Test vs ‘Ortice’ Test condition; 3: ‘Ortice’ Test vs ‘Ortice’ Control condition; 4: ‘Ruveia’ Control vs ‘Ortice’ Control condition. Gradation from green to red is relative to the log2 Fold Change (FC) values. Similarities were calculated using Euclidean distances and agglomeration was performed according to the complete-linkage algorithm.
(TIF)

S5 Fig Real time RT-PCR validation of the microarray results. The expression level of four DEGs (1, 2, 3 and 4) and two transcripts that were not affected by *B*. *oleae* (5 and 6) was analysed by real-time RT-PCR in drupes of the ‘Ortice’ cultivar. Quantities are reported on a linear scale relative to the calibrator condition (undamaged olives). 1: G0MWCVW03GE3QK; 2: G0MWCVW01A1C2H; 3: G0MWCVW03FSPSU; 4: G0MWCVW04JNBZP; 5: contig04878; 6: G0MWCVW02DVZFG. See S1 Table for details on the transcripts. For each transcript, an asterisk indicates a significant difference with the control condition (Student t-test; *: p<0.05).
(TIF)

S6 Fig Level 2 chart summary of GO term association for the overexpressed (orange bars) and under expressed (blue bars) sequences following *B*. *oleae* feeding of the ‘Ruveia’ drupes. (TIF)

S7 Fig Level 2 chart summary of GO term association for the up-regulated (orange bars) and down-regulated (blue bars) sequences of the comparison between infested drupes of the ‘Ruveia’ and ‘Ortice’ varieties. (TIF)

S1 Table Primer used for qRT-PCR validation of the microrray results. (XLSX)

S2 Table List of all unigenes and their predicted functional annotation. (XLSX)

S3 Table K-means clusters of the differentially expressed genes. (XLSX)

S1 File Differentially expressed genes and their annotation. Table A. List and annotation of the underexpressed genes (FDR: <0.05; log2Ratio <1) between attacked drupes of the Ruveia and attacked drupes of the Ortice cultivar. Table B. List and annotation of the overexpressed genes (FDR: <0.05; log2Ratio >1) between attacked drupes of the Ruveia and the attacked drupes of the Ortice cultivar. Table C. List and annotation of the underexpressed genes (FDR: <0.05; log2Ratio <1) between attacked and control drupes of the Ruveia cultivar. Table D. List and annotation of the overexpressed genes (FDR: <0.05; log2Ratio >1) between attacked and control drupes of the Ruveia cultivar. Table E. List and annotation of the underexpressed genes (FDR: <0.05; log2Ratio <1) between attacked and control drupes of the Ortice cultivar. F. List and annotation of the overexpressed genes (FDR: <0.05; log2Ratio >1) between attacked and control drupes of the Ortice cultivar. Table G. List and annotation of the underexpressed genes (FDR: <0.05; log2Ratio <1) between control drupes of the Ruveia and control drupes of the Ortice cultivar. Table H. List and annotation of the overexpressed genes (FDR: <0.05; log2Ratio >1) between control drupes of the Ruveia and control drupes of the Ortice cultivar.
(XLSX)

## References

[1] Nardi F, Carapelli A, Boore J, Roderick G, Dallai R, Frati F. Domestication of olive fly through a multi-regional host shift to cultivated olives: Comparative dating using complete mitochondrial genomes. Molecular phylogenetics and evolution. 2010;57(2):678–86. doi: 10.1016/j.ympev.2010.08.008 20723608 10.1016/j.ympev.2010.08.008

[2] Zygouridis N, Augustinos A, Zalom F, Mathiopoulos K. Analysis of olive fly invasion in California based on microsatellite markers. Heredity. 2009;102(4):402–12. doi: 10.1038/hdy.2008.125 19107137 10.1038/hdy.2008.125

[3] Fletcher B. The biology of dacine fruit flies. Annual review of entomology. 1987;32(1):115–44.

[4] Daane KM, Johnson MW. Olive fruit fly: managing an ancient pest in modern times. Annual review of entomology. 2010;55:151–69. doi: 10.1146/annurev.ento.54.110807.090553 19961328 10.1146/annurev.ento.54.110807.090553

[5] Malheiro R, Casal S, Baptista P, Pereira JA. A review of *Bactrocera oleae* (Rossi) impact in olive products: From the tree to the table. Trends in Food Science & Technology. 2015;44(2):226–42.

[6] Gómez-Caravaca AM, Cerretani L, Bendini A, Segura-Carretero A, Fernández-Gutiérrez A, Del Carlo M, et al Effects of fly attack (*Bactrocera oleae*) on the phenolic profile and selected chemical parameters of olive oil. Journal of Agricultural and Food Chemistry. 2008;56(12):4577–83. doi: 10.1021/jf800118t 18522402 10.1021/jf800118t

[7] Gucci R, Caruso G, Canale A, Loni A, Raspi A, Urbani S, et al Qualitative changes of olive oils obtained from fruits damaged by *Bactrocera oleae* (Rossi). HortScience. 2012;47(2):301–6.

[8] Angerosa F, Giacinto LD, Solinas M. Influence of *Dacus oleae* infestation on flavor of oils, extracted from attacked olive fruits, by HPLC and HRGC analyses of volatile compounds. Grasas y Aceites. 1992;43(3):134–42.

[9] Medjkouh L, Tamendjari A, Keciri S, Santos J, Nunes MA, Oliveira M. The effect of the olive fruit fly (*Bactrocera oleae*) on quality parameters, and antioxidant and antibacterial activities of olive oil. Food & Function. 2016;7(6):2780–8. 27220688 10.1039/c6fo00295a

[10] Vontas J, Hejazi M, Hawkes NJ, Cosmidis N, Loukas M, Hemingway J. Resistance-associated point mutations of organophosphate insensitive acetylcholinesterase, in the olive fruit fly *Bactrocera oleae*. Insect molecular biology. 2002;11(4):329–36. 12144698 10.1046/j.1365-2583.2002.00343.x

[11] Tsolakis H, Ragusa E, Tarantino P. Control of *Bactrocera oleae* by low environmental impact methods: NPC methodology to evaluate the efficacy of lure-and-kill method and copper hydroxide treatments. Bulletin of Insectology. 2011;64(1):1–8.

[12] Daane KM, Wang X, Nieto DJ, Pickett CH, Hoelmer KA, Blanchet A, et al Classic biological control of olive fruit fly in California, USA: release and recovery of introduced parasitoids. BioControl. 2015;60(3):317–30.

[13] Hoelmer KA, Kirk AA, Pickett CH, Daane KM, Johnson MW. Prospects for improving biological control of olive fruit fly, *Bactrocera oleae* (Diptera: Tephritidae), with introduced parasitoids (Hymenoptera). Biocontrol science and technology. 2011;21(9):1005–25.

[14] Corrado G, Alagna F, Rocco M, Renzone G, Varricchio P, Coppola V, et al Molecular interactions between the olive and the fruit fly *Bactrocera oleae*. BMC Plant Biology. 2012;12(1):1. 22694925 10.1186/1471-2229-12-86 PMC3733423

[15] Alagna F, Kallenbach M, Pompa A, De Marchis F, Rao R, Baldwin IT, et al Olive fruits infested with olive fly larvae respond with an ethylene burst and the emission of specific volatiles. Journal of Integrative Plant Biology. 2015. 10.1111/jipb.12343 25727685

[16] Iannotta N, Scalercio S. Susceptibility of Cultivars to Biotic Stresses In: Mazzalupo I, editor. Olive Germplasm—The Olive Cultivation, Table Olive and Olive Oil Industry in Italy: INTECH Open Access Publisher; 2012 p. 81–106.

[17] Rizzo R, Caleca V, editors. Resistance to the attack of Bactrocera oleae (Gmelin) of some Sicilian olive cultivars. Proceedings of Olivebioteq 2006, Second International Seminar “Biotechnology and quality of olive tree products around the Mediterranean Basin” November 5th–10th, Mazara del Vallo, Marsala, Italy; 2006.

[18] Di Vaio C. Il germoplasma dell'olivo in Campania. Napoli (IT): Assessorato all'Agricoltura Regione Campania; 2012 92 p.

[19] Pugliano G. La risorsa genetica dell'olivo in Campania. Napoli (IT): Regione Campania; 2000 158 p.

[20] Corrado G, Garonna A, Cabanás CG-L, Gregoriou M, Martelli GP, Mathiopoulos KD, et al Host Response to Biotic Stresses In: Rugini E, Baldoni L, Muleo R, Sebastiani L, editors. The Olive Tree Genome. Springer International Publishing; 2016 p. 75–98.

[21] Malheiro R, Casal S, Cunha SC, Baptista P, Pereira JA. Identification of leaf volatiles from olive (*Olea europaea*) and their possible role in the ovipositional preferences of olive fly, *Bactrocera oleae* (Rossi)(Diptera: Tephritidae). Phytochemistry. 2016;121:11–9. doi: 10.1016/j.phytochem.2015.10.005 26603276 10.1016/j.phytochem.2015.10.005

[22] Lo Scalzo RL, Scarpati ML, Verzegnassi B, Vita G. *Olea europaea* chemicals repellent to *Dacus oleae* females. Journal of Chemical Ecology. 1994;20(8):1813–23. doi: 10.1007/BF02066224 24242710 10.1007/BF02066224

[23] Garantonakis N, Varikou K, Markakis E, Birouraki A, Sergentani C, Psarras G, et al Interaction between *Bactrocera oleae* (Diptera: Tephritidae) infestation and fruit mineral element content in *Olea europaea* (Lamiales: Oleaceae) cultivars of global interest. Applied Entomology and Zoology. 2016;51(2):257–65.

[24] Scarpati ML, Lo Scalzo RL, Vita G, Gambacorta A. Chemiotropic behavior of female olive fly (Bactrocera oleae Gmel.) on *Olea europaea* L. Journal of Chemical Ecology. 1996;22(5):1027–36. doi: 10.1007/BF02029952 24227622 10.1007/BF02029952

[25] Rizzo R, Caleca V, Lombardo A. Relation of fruit color, elongation, hardness, and volume to the infestation of olive cultivars by the olive fruit fly, *Bactrocera oleae*. Entomologia Experimentalis et Applicata. 2012;145(1):15–22.

[26] Uceda M, Frias L. Harvest dates Evolution of the fruit oil content, oil composition and oil quality. Proc II Seminario Oleicola Internacional, Córdoba (Spain), 1975:6–17.

[27] Alagna F, D'Agostino N, Torchia L, Servili M, Rao R, Pietrella M, et al Comparative 454 pyrosequencing of transcripts from two olive genotypes during fruit development. BMC Genomics. 2009;10(1):1. 19709400 10.1186/1471-2164-10-399 PMC2748093

[28] Coppola V, Coppola M, Rocco M, Digilio MC, D’Ambrosio C, Renzone G, et al Transcriptomic and proteomic analysis of a compatible tomato-aphid interaction reveals a predominant salicylic acid-dependent plant response. BMC Genomics. 2013;14(1):1. 23895395 10.1186/1471-2164-14-515 PMC3733717

[29] R Developmental Team Core. R: A language and environment for statistical computing: R Foundation for Statistical Computing; 2009.

[30] Voigt C. Synthetic Biology: Methods for part/device characterization and chassis engineering: Academic Press; 2011.

[31] Saeed A, Sharov V, White J, Li J, Liang W, Bhagabati N, et al TM4: a free, open-source system for microarray data management and analysis. Biotechniques. 2003;34(2):374 12613259 10.2144/03342mt01

[32] Zimmermann P, Schildknecht B, Craigon D, Garcia-Hernandez M, Gruissem W, May S, et al MIAME/Plant—adding value to plant microarrray experiments. Plant Methods. 2006;2(1):1. 16401339 10.1186/1746-4811-2-1 PMC1334190

[33] Götz S, García-Gómez JM, Terol J, Williams TD, Nagaraj SH, Nueda MJ, et al High-throughput functional annotation and data mining with the Blast2GO suite. Nucleic Acids Research. 2008;36(10):3420–35. doi: 10.1093/nar/gkn176 18445632 10.1093/nar/gkn176 PMC2425479

[34] Myhre S, Tveit H, Mollestad T, Lægreid A. Additional gene ontology structure for improved biological reasoning. Bioinformatics. 2006;22(16):2020–7. doi: 10.1093/bioinformatics/btl334 16787968 10.1093/bioinformatics/btl334

[35] Muñoz-Mérida A, Viguera E, Claros MG, Trelles O, Pérez-Pulido AJ. Sma3s: a three-step modular annotator for large sequence datasets. DNA research. 2014;21(4):341–53. doi: 10.1093/dnares/dsu001 24501397 10.1093/dnares/dsu001 PMC4131829

[36] Selote D, Kachroo A. RIN4-like proteins mediate resistance protein-derived soybean defense against *Pseudomonas syringae*. Plant Signaling & Behavior. 2010;5(11):1453–6. 21051954 10.4161/psb.5.11.13462 PMC3115253

[37] Koudounas K, Banilas G, Michaelidis C, Demoliou C, Rigas S, Hatzopoulos P. A defence-related *Olea europaea* β-glucosidase hydrolyses and activates oleuropein into a potent protein cross-linking agent. Journal of Experimental Botany. 2015;66(7):2093–106. doi: 10.1093/jxb/erv002 25697790 10.1093/jxb/erv002 PMC4669557

[38] Liu H, Wang X, Zhang H, Yang Y, Ge X, Song F. A rice serine carboxypeptidase-like gene OsBISCPL1 is involved in regulation of defense responses against biotic and oxidative stress. Gene. 2008;420(1):57–65. doi: 10.1016/j.gene.2008.05.006 18571878 10.1016/j.gene.2008.05.006

[39] Moura DS, Bergey DR, Ryan CA. Characterization and localization of a wound-inducible type I serine-carboxypeptidase from leaves of tomato plants (*Lycopersicon esculentum* Mill.). Planta. 2001;212(2):222–30. doi: 10.1007/s004250000380 11216843 10.1007/s004250000380

[40] Kafer C, Zhou L, Santoso D, Guirgis A, Weers B, Park S, et al Regulation of pyrimidine metabolism in plants. Front Biosci. 2004;9:1611–25. 14977572 10.2741/1349

[41] Muñoz-Mérida A, González-Plaza JJ, Blanco AM, del Carmen García-López M, Rodríguez JM, Pedrola L, et al De novo assembly and functional annotation of the olive (*Olea europaea*) transcriptome. DNA research. 2013;20(1):93–108. doi: 10.1093/dnares/dss036 23297299 10.1093/dnares/dss036 PMC3576661

[42] Thudi M, Li Y, Jackson SA, May GD, Varshney RK. Current state-of-art of sequencing technologies for plant genomics research. Briefings in Functional Genomics. 2012;11(1):3–11. doi: 10.1093/bfgp/elr045 22345601 10.1093/bfgp/elr045

[43] Ozdemir Ozgenturk N, Oruç F, Sezerman U, Kuçukural A, Vural Korkut S, Toksoz F, et al Generation and analysis of expressed sequence tags from *Olea europaea* L. Comparative and functional genomics. 2010;2010. 10.1155/2010/757512 PMC3004401 21197085

[44] Armstead I, Huang L, Ravagnani A, Robson P, Ougham H. Bioinformatics in the orphan crops. Briefings in bioinformatics. 2009:bbp036. 10.1093/bib/bbp036 19734255

[45] García-López MC, Vidoy I, Jiménez-Ruiz J, Muñoz-Mérida A, Fernández-Ocaña A, de la Rosa R, et al Genetic changes involved in the juvenile-to-adult transition in the shoot apex of *Olea europaea* L. occur years before the first flowering. Tree Genetics & Genomes. 2014;10(3):585–603.

[46] González-Plaza JJ, Ortiz-Martín I, Muñoz-Mérida A, García-López C, Sánchez-Sevilla JF, Luque F, et al Transcriptomic analysis using olive varieties and breeding progenies identifies candidate genes involved in plant architecture. Frontiers in Plant Science. 2016;7. 26973682 10.3389/fpls.2016.00240 PMC4773642

[47] Schuman MC, Heinzel N, Gaquerel E, Svatos A, Baldwin IT. Polymorphism in jasmonate signaling partially accounts for the variety of volatiles produced by *Nicotiana attenuata* plants in a native population. New Phytologist. 2009;183(4):1134–48. doi: 10.1111/j.1469-8137.2009.02894.x 19538549 10.1111/j.1469-8137.2009.02894.x

[48] Wu J, Hettenhausen C, Schuman MC, Baldwin IT. A comparison of two *Nicotiana attenuata* accessions reveals large differences in signaling induced by oral secretions of the specialist herbivore Manduca sexta. Plant Physiology. 2008;146(3):927–39. doi: 10.1104/pp.107.114785 18218965 10.1104/pp.107.114785 PMC2259078

[49] Kliebenstein DJ, Kroymann J, Brown P, Figuth A, Pedersen D, Gershenzon J, et al Genetic control of natural variation in Arabidopsis glucosinolate accumulation. Plant Physiology. 2001;126(2):811–25. doi: 10.1104/pp.126.2.811 11402209 10.1104/pp.126.2.811 PMC111171

[50] Windsor AJ, Reichelt M, Figuth A, Svatoš A, Kroymann J, Kliebenstein DJ, et al Geographic and evolutionary diversification of glucosinolates among near relatives of *Arabidopsis thaliana* (Brassicaceae). Phytochemistry. 2005;66(11):1321–33. doi: 10.1016/j.phytochem.2005.04.016 15913672 10.1016/j.phytochem.2005.04.016

[51] Broekgaarden C, Poelman EH, Steenhuis G, Voorrips RE, Dicke M, Vosman B. Responses of *Brassica oleracea* cultivars to infestation by the aphid *Brevicoryne brassicae*: an ecological and molecular approach. Plant, cell & environment. 2008;31(11):1592–605. 10.1111/j.1365-3040.2008.01871.x 18721268

[52] Grasso F, Paduano A, Corrado G, Ambrosino ML, Rao R, Sacchi R. DNA diversity in olive (*Olea europaea L*.) and its relationships with fatty acid, biophenol and sensory profiles of extra virgin olive oils. Food Research International. 2016:86:121–130.

[53] Kliebenstein DJ, Figuth A, Mitchell-Olds T. Genetic architecture of plastic methyl jasmonate responses in *Arabidopsis thaliana*. Genetics. 2002;161(4):1685–96. 12196411 10.1093/genetics/161.4.1685 PMC1462221

[54] Sauge MH, Mus F, Lacroze JP, Pascal T, Kervella J, Poëssel JL. Genotypic variation in induced resistance and induced susceptibility in the peach-*Myzus persicae* aphid system. Oikos. 2006;113(2):305–13.

[55] Walling LL. Avoiding effective defenses: strategies employed by phloem-feeding insects. Plant Physiology. 2008;146(3):859–66. doi: 10.1104/pp.107.113142 18316641 10.1104/pp.107.113142 PMC2259051

[56] Pieterse CM, Dicke M. Plant interactions with microbes and insects: from molecular mechanisms to ecology. Trends in Plant Science. 2007;12(12):564–9. doi: 10.1016/j.tplants.2007.09.004 17997347 10.1016/j.tplants.2007.09.004

[57] Musser RO, Hum-Musser SM, Eichenseer H, Peiffer M, Ervin G, Murphy JB, et al Herbivory: caterpillar saliva beats plant defences. Nature. 2002;416(6881):599–600. doi: 10.1038/416599a 11948341 10.1038/416599a

[58] Sarmento RA, Lemos F, Bleeker PM, Schuurink RC, Pallini A, Oliveira MGA, et al A herbivore that manipulates plant defence. Ecology letters. 2011;14(3):229–36. doi: 10.1111/j.1461-0248.2010.01575.x 21299823 10.1111/j.1461-0248.2010.01575.x PMC3084520

[59] Glas JJ, Alba JM, Simoni S, Villarroel CA, Stoops M, Schimmel BC, et al Defense suppression benefits herbivores that have a monopoly on their feeding site but can backfire within natural communities. BMC biology. 2014;12(1):98. 25403155 10.1186/s12915-014-0098-9 PMC4258945

[60] Tayeh C, Randoux B, Tisserant B, Khong G, Jacques P, Reignault P. Are ineffective defence reactions potential target for induced resistance during the compatible wheat-powdery mildew interaction? Plant Physiology and Biochemistry. 2015;96:9–19. doi: 10.1016/j.plaphy.2015.07.015 26218548 10.1016/j.plaphy.2015.07.015

[61] Wu J, Baldwin IT. New insights into plant responses to the attack from insect herbivores. Annual review of genetics. 2010;44:1–24. doi: 10.1146/annurev-genet-102209-163500 20649414 10.1146/annurev-genet-102209-163500

[62] Benitez Y, Botella MA, Trapero A, Alsalimiya M, Caballero JL, Dorado G, et al Molecular analysis of the interaction between *Olea europaea* and the biotrophic fungus *Spilocaea oleagina*. Molecular plant pathology. 2005;6(4):425–38. doi: 10.1111/j.1364-3703.2005.00290.x 20565668 10.1111/j.1364-3703.2005.00290.x

[63] Zhou S, Lou Y-R, Tzin V, Jander G. Alteration of plant primary metabolism in response to insect herbivory. Plant physiology. 2015;169(3):1488–98. doi: 10.1104/pp.15.01405 26378101 10.1104/pp.15.01405 PMC4634104

[64] Martinelli F, Remorini D, Saia S, Massai R, Tonutti P. Metabolic profiling of ripe olive fruit in response to moderate water stress. Scientia Horticulturae. 2013;159:52–8.

[65] La Camera S, Gouzerh G, Dhondt S, Hoffmann L, Fritig B, Legrand M, et al Metabolic reprogramming in plant innate immunity: the contributions of phenylpropanoid and oxylipin pathways. Immunological reviews. 2004;198(1):267–84. 15199968 10.1111/j.0105-2896.2004.0129.x

[66] Rossi L, Borghi M, Francini A, Lin X, Xie D-Y, Sebastiani L. Salt stress induces differential regulation of the phenylpropanoid pathway in *Olea europaea* cultivars Frantoio (salt-tolerant) and Leccino (salt-sensitive). Journal of Plant Physiology. 2016;204:8–15. doi: 10.1016/j.jplph.2016.07.014 27497740 10.1016/j.jplph.2016.07.014

