# Supplementary figures and images for: The transcriptional response to the olive fruit fly (Bactrocera oleae) reveals extended differences between tolerant and susceptible olive (Olea europaea L.) varieties
Source: PLoS One. 2017 Aug 10;12(8):e0183050. doi: 10.1371/journal.pone.0183050 (PMC5552259; doi:10.1371/journal.pone.0183050)

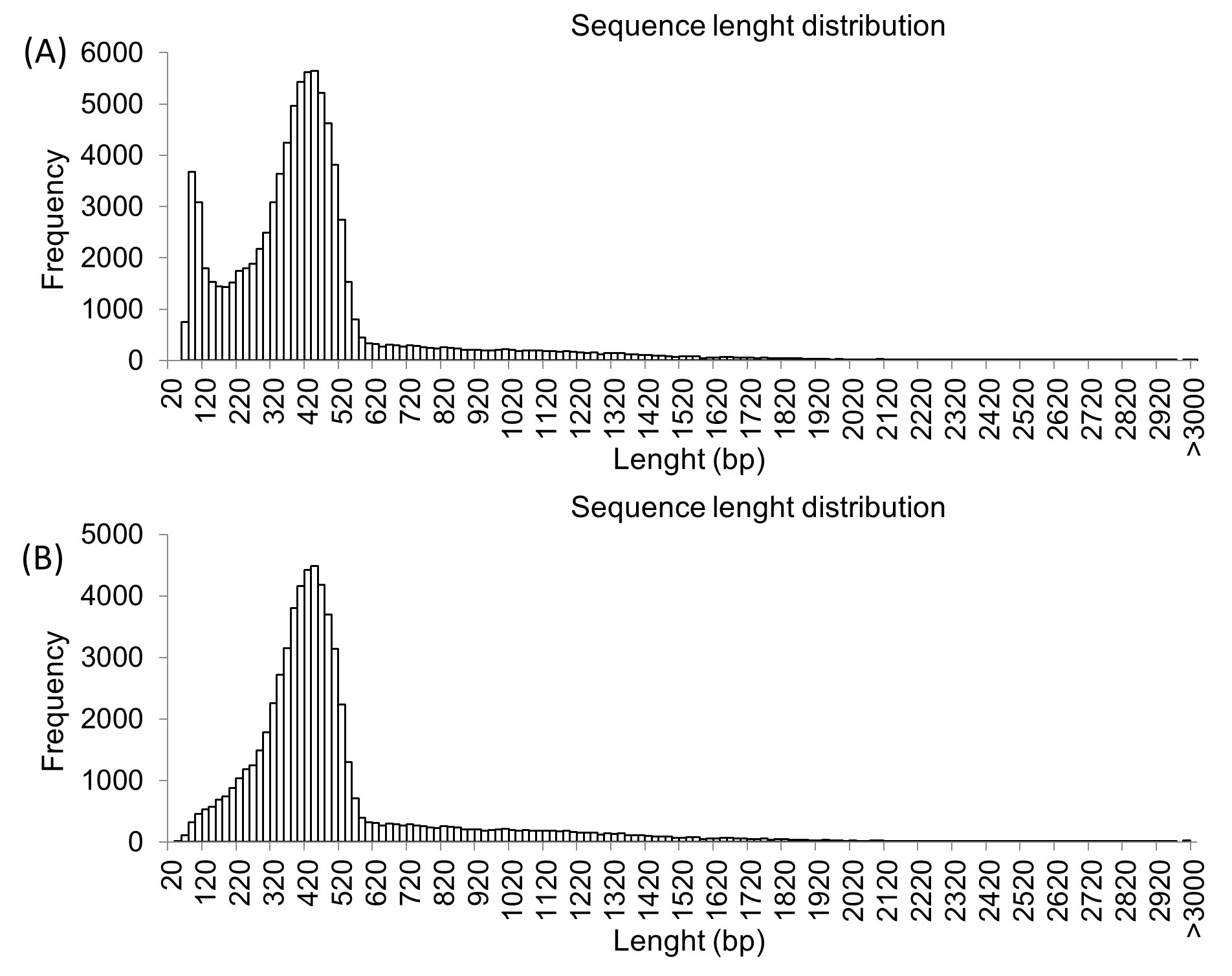

Supplement: S1 Fig — (TIF) [file pone.0183050.s001.tif]

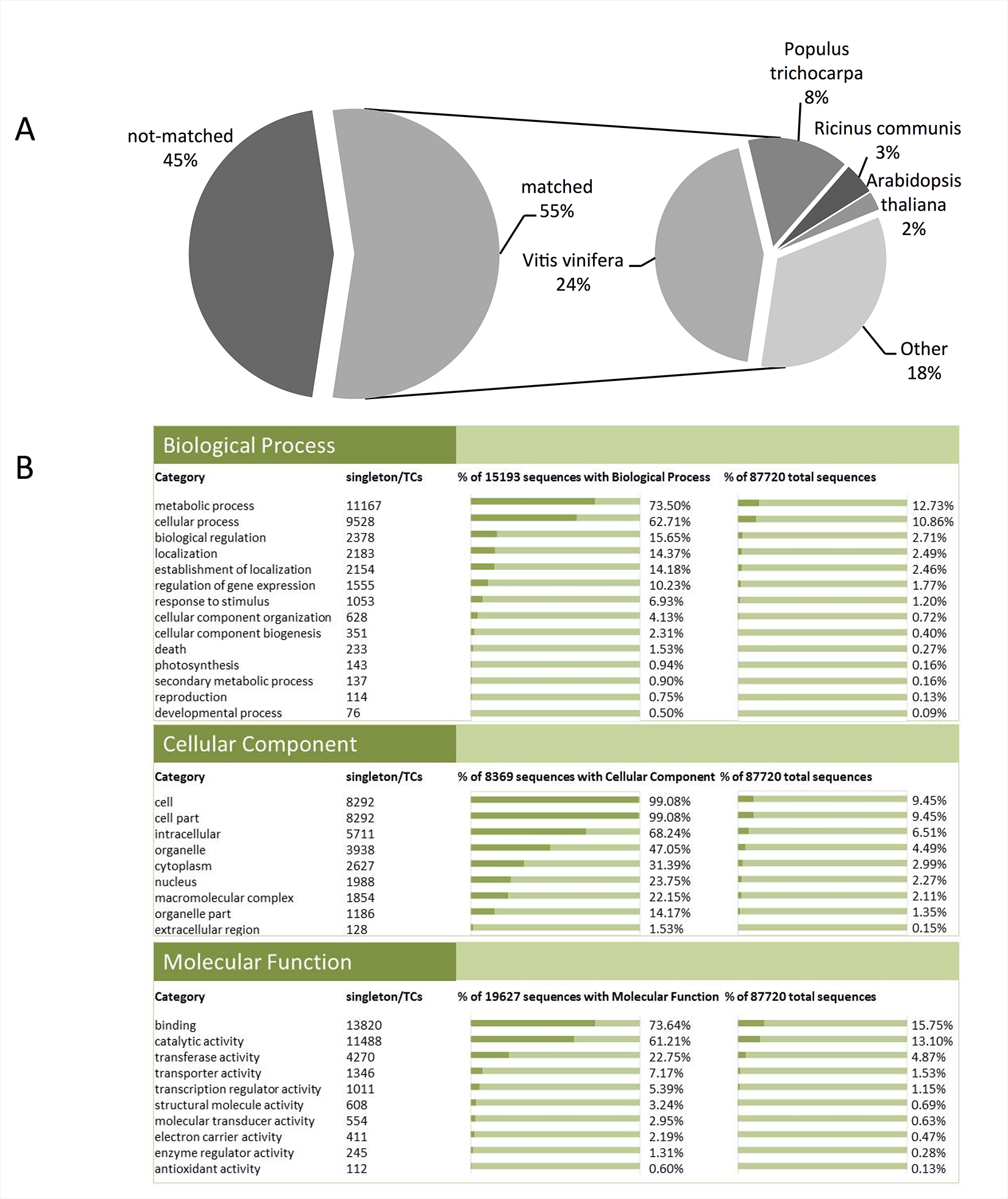

Supplement: S2 Fig — A) Distribution of the unigenes according to the presence (matched) or absence (non-matched) of hits retrieved from the NCBI database. The pie of the pie chart illustrates the relative distribution of the best-BlastX hits according to the plant species. B) Classification of the unigenes in the GO domains Biological Process, Cellular Component and Molecular Function. For each domain, the table reports the GO-terms (category) ranked in decreasing order according to the number of sequences (singleton/TCs). The bar chart illustrates for each GO-term the relative amount in relation to the number of sequences annotated in the GO-domain and the total number of sequences assembled. (TIFF) [file pone.0183050.s002.tiff]

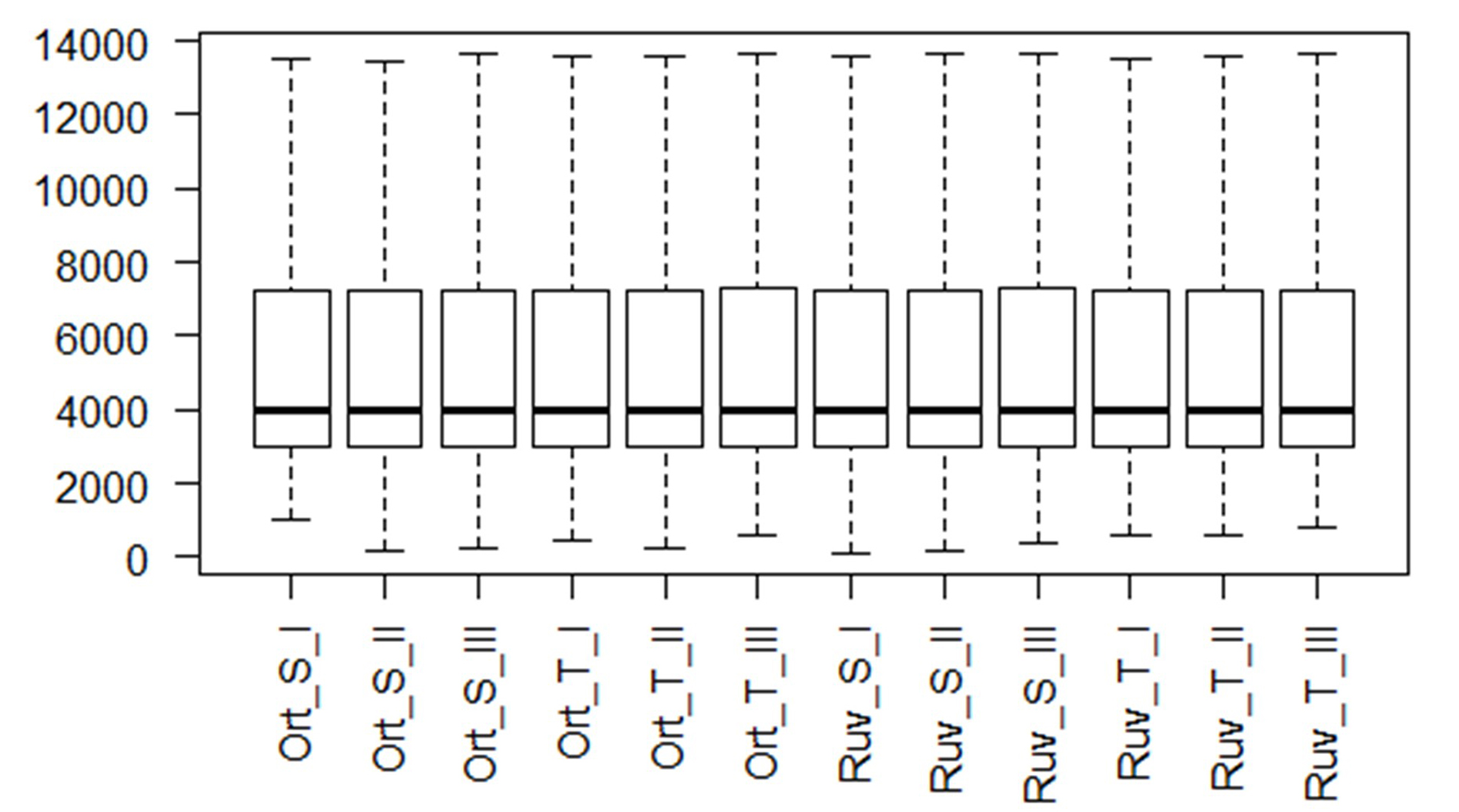

Supplement: S3 Fig — Legend: Ort: ‘Ortice’; Ruv: ‘Ruveia’; S: control condition; T: test condition; Roman numbers denote, per each condition, the biological replicate. (TIF) [file pone.0183050.s003.tif]

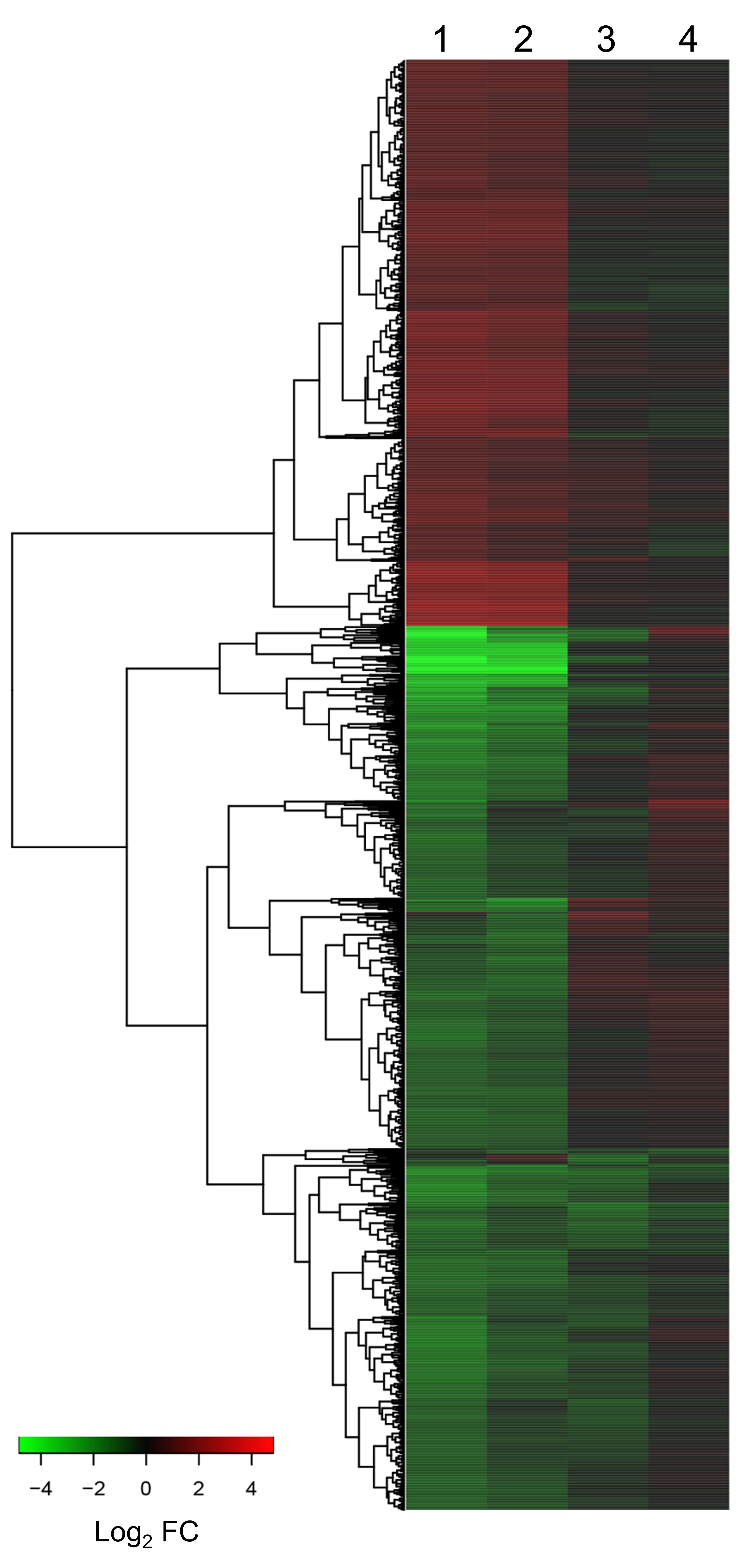

Supplement: S4 Fig — The heatmap shows the relative expression level of the DEGs in the four experimental comparisons. 1: ‘Ruveia’ Test vs ‘Ruveia’ Control condition; 2: ‘Ruveia’ Test vs ‘Ortice’ Test condition; 3: ‘Ortice’ Test vs ‘Ortice’ Control condition; 4: ‘Ruveia’ Control vs ‘Ortice’ Control condition. Gradation from green to red is relative to the log2 Fold Change (FC) values. Similarities were calculated using Euclidean distances and agglomeration was performed according to the complete-linkage algorithm. (TIF) [file pone.0183050.s004.tif]

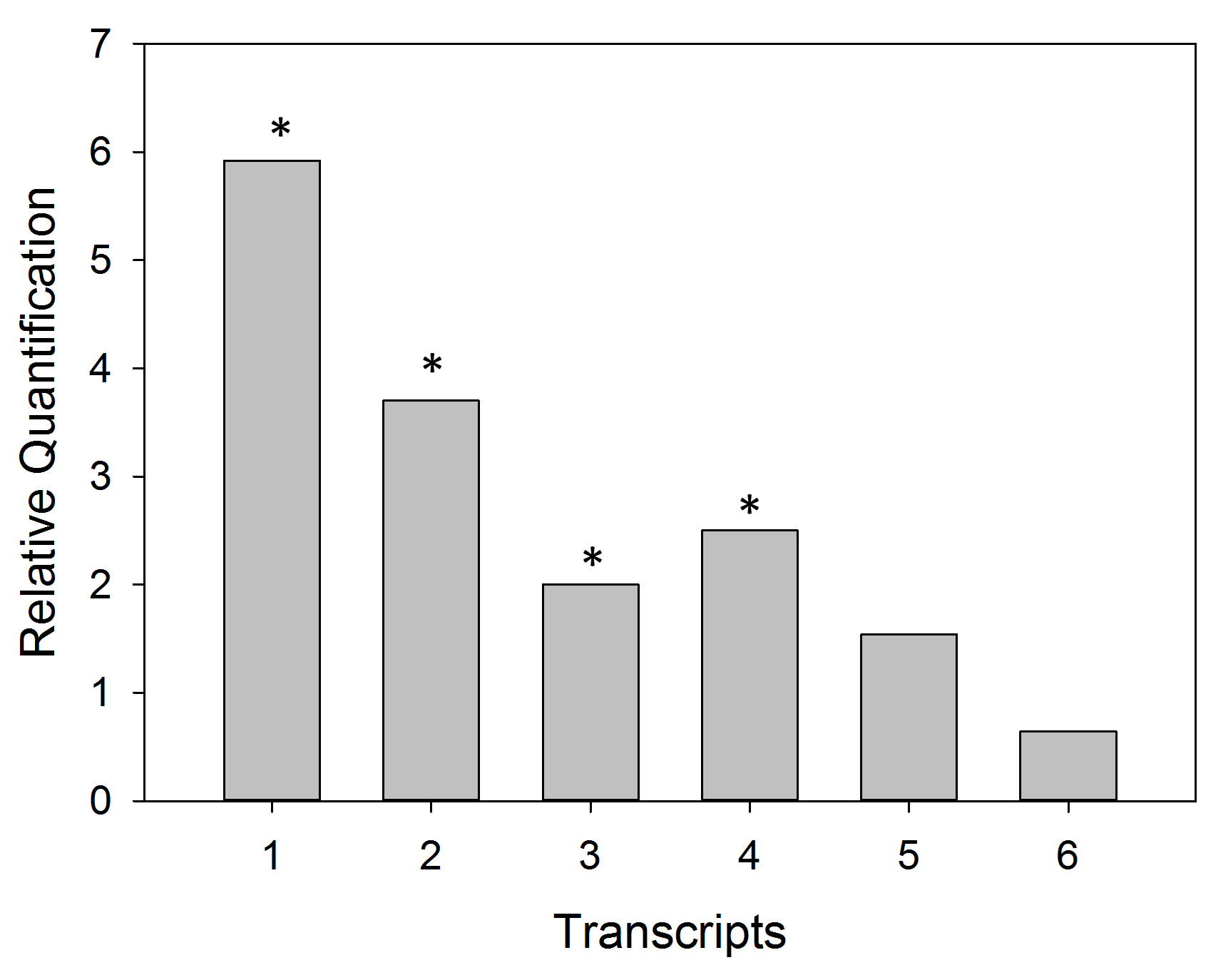

Supplement: S5 Fig — The expression level of four DEGs (1, 2, 3 and 4) and two transcripts that were not affected by B. oleae (5 and 6) was analysed by real-time RT-PCR in drupes of the ‘Ortice’ cultivar. Quantities are reported on a linear scale relative to the calibrator condition (undamaged olives). 1: G0MWCVW03GE3QK; 2: G0MWCVW01A1C2H; 3: G0MWCVW03FSPSU; 4: G0MWCVW04JNBZP; 5: contig04878; 6: G0MWCVW02DVZFG. See S1 Table for details on the transcripts. For each transcript, an asterisk indicates a significant difference with the control condition (Student t-test; *: p<0.05). (TIF) [file pone.0183050.s005.tif]

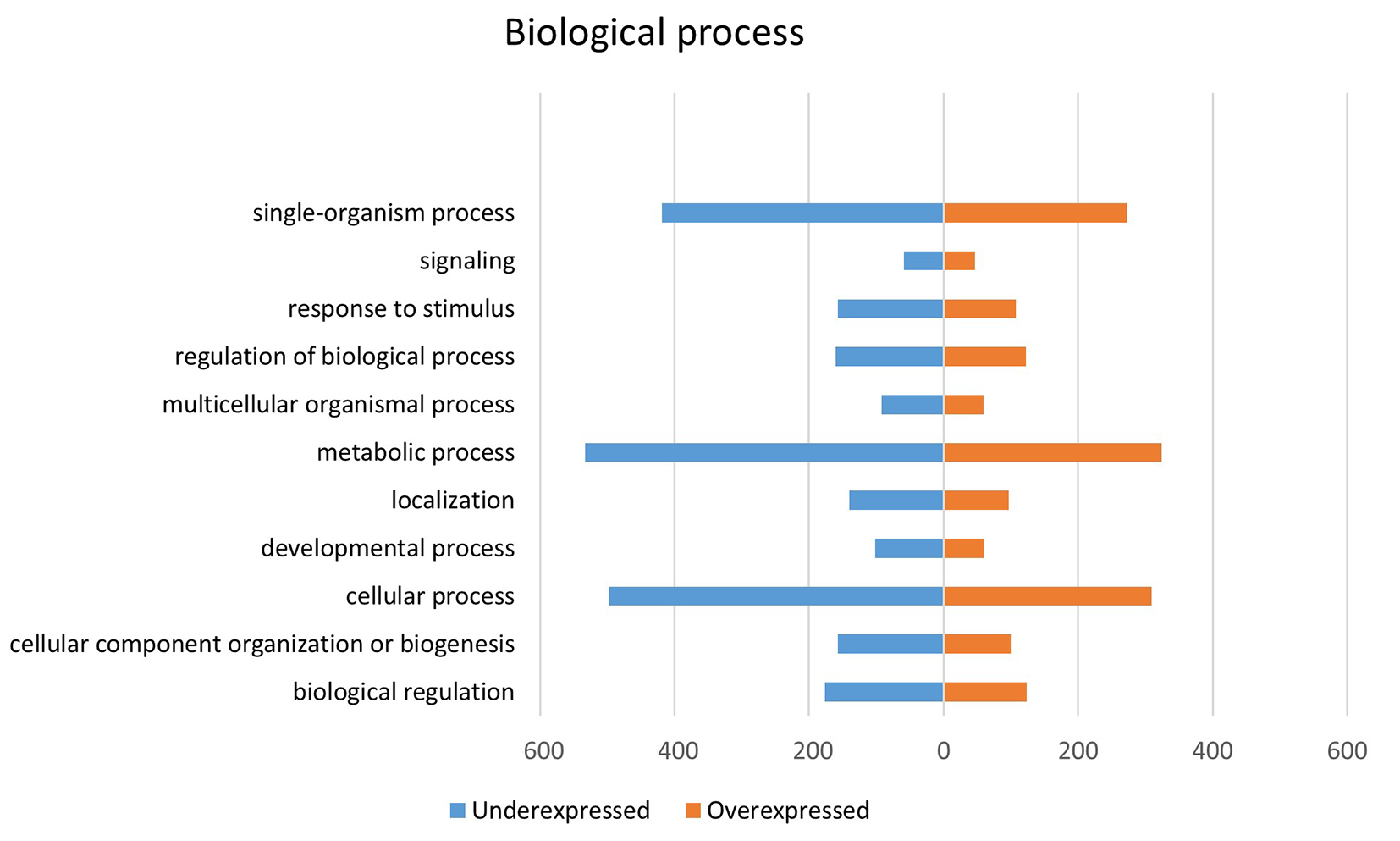

Supplement: S6 Fig — (TIF) [file pone.0183050.s006.tif]

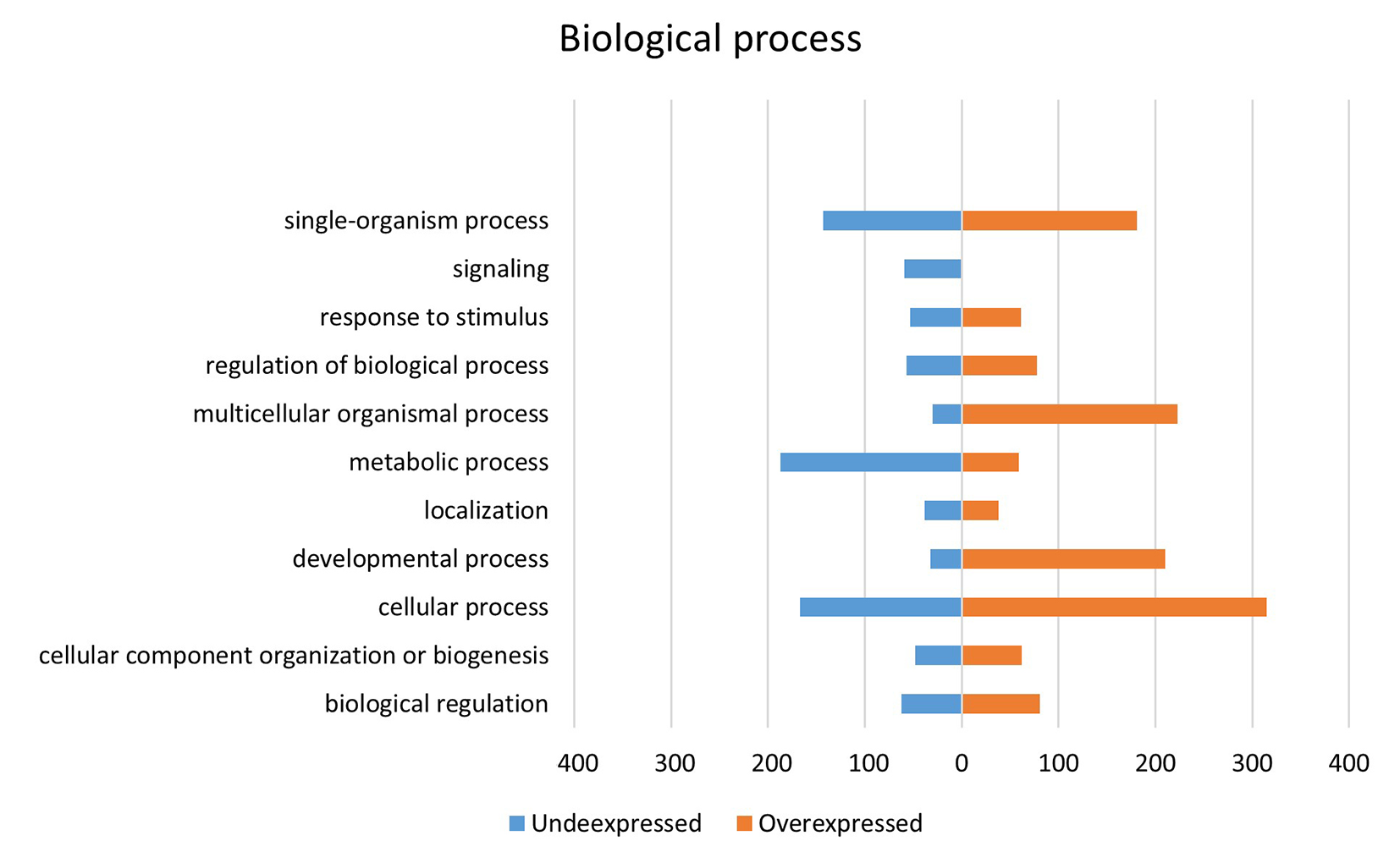

Supplement: S7 Fig — (TIF) [file pone.0183050.s007.tif]
